# Simultaneous detection of gradual and abrupt structural changes in Bayesian longitudinal modelling using entropy and model fit measures

**DOI:** 10.1111/bmsp.70029

**Published:** 2026-01-07

**Authors:** Yanling Li, Xiaoyue Xiong, Zita Oravecz, Sy‐Miin Chow

**Affiliations:** ^1^ Department of Human Development and Family Studies Pennsylvania State University University Park Pennsylvania USA; ^2^ Social Science Research Institute Pennsylvania State University University Park Pennsylvania USA; ^3^ Institute for Computational and Data Sciences Pennsylvania State University University Park Pennsylvania USA

**Keywords:** growth curve, mHealth intervention, model selection, psychological well‐being, regime‐switching, time‐varying parameter

## Abstract

Although individuals may exhibit both gradual and abrupt changes in their dynamic properties as shaped by both slowly accumulating influences and acute events, existing statistical frameworks offer limited capacity for the simultaneous detection and representation of these distinct change patterns. We propose a Bayesian regime‐switching (RS) modelling framework and an entropy measure adapted from the frequentist framework to facilitate simultaneous representation and testing of postulates of gradual and abrupt changes. Results from Monte Carlo simulation studies indicated that using a combination of entropy and information criterion measures such as the Bayesian information criterion was consistently most effective at facilitating the selection of the best‐fitting model across varying magnitudes of abrupt changes. We found that slight lower entropy thresholds may be helpful in facilitating the selection of longitudinal models with RS properties as this class of models tended to yield lower entropy values than conventional thresholds for reliable classification in cross‐sectional mixture models—even under satisfactory parameter recovery and classification results. We fitted the proposed models and other candidate models to the data collected from an intervention study on the psychological well‐being (PWB) of college‐attending early adults. Results suggested abrupt, regime‐related transitions in the intra‐individual variability levels of PWB dynamics among some participants following the intervention period. Practical usage of the entropy measure in conjunction with other model selection measures, and guidelines to enhance simultaneous detection of true abrupt and gradual changes are discussed.

## 1 INTRODUCTION

Psychological processes may display changes that alter their dynamic characteristics (e.g. means, variances) (Fox, 1972; Haan‐Rietdijk et al., 2016; Nelson & Plosser, 1982; Perron, 1989). For instance, an intervention may affect individuals' positive emotion dynamics in multiple ways, such as gradually improving their emotion regulation ability to enable quicker recovery from external disturbance (e.g. negative events, stressful experiences) or elevating their baseline (“trait”) levels of positive emotion (Kuppens et al., 2010; Segrin & Taylor, 2007). Instead of transitory fluctuations at isolated time points, such changes are characterized by persistent alterations in the dynamic and measurement characteristics of a system and are referred to as *structural changes* in this article.

Examples of dynamic‐related structural changes include changes in the autocorrelation coefficient that controls how rapidly an individual's emotion returns to or deviates from the individual's true (latent) baseline in the face of external perturbations. Examples of measurement‐related structural changes include changes to the intercepts, factor loading and measurement error variance structures of a system. Because some dynamic and measurement parameters, with appropriate constraints, may be expressed as reparameterizations of each other (e.g., simplex processes may be represented as factor analytic models; Chow et al., 2010), we distinguish structural from other transitory changes in terms of their sustainability over time, as opposed to changes in a particular type of parameters. Regardless of whether the changes affect dynamic or measurement‐related parameters, they must persist for a period of time to be considered structural changes. Thus, an unusually high test score that lasts one occasion and that occasion only is not regarded as a structural change, whereas a shift in latent ability that is reflected in improved test scores over multiple occasions is considered a structural change.

Structural changes can, in turn, be categorized into abrupt and gradual changes. Conceptually, a gradual change refers to a slow, smooth and less noticeable shift in the value of a construct over a long period of time, whereas an abrupt change is a sudden and usually discontinuous shift. The present article focuses on detecting structural changes related to the means and variances of autocorrelated processes, as well as individual differences in these changes.

The past decades have seen a marked growth in statistical approaches developed for detecting structural changes (e.g., Chow et al., 2011; Fahrmeir et al., 2004; Fan & Zhang, 2008; Heiby, 1995; Hoover et al., 1998; Huang et al., 2004; Molenaar et al., 1992). Despite their increased popularity as a representation of change, existing methods for detecting structural changes tend to focus on gradual or abrupt changes, but not both. The few exceptions that do (Hayes et al., 2007; Thomas & Persons, 2013; Vittengl et al., 2015) have capitalized on descriptive statistics or visualization approaches to make inferences about gradual and abrupt changes. As models aimed at capturing gradual and/or abrupt changes increase in complexity, so is the need for model selection approaches that can help determine whether inclusion of either or both types of changes in a model is warranted.

In this study, we introduce and evaluate a modelling framework that combines time‐varying parameter (TVP) models with regime‐switching (RS) models. This integrated approach enables simultaneous investigation of gradual (via TVP) and abrupt changes (via RS) in dynamic characteristics, as well as individual differences in these changes. We also evaluate the effectiveness of several model selection measures, including a new Bayesian entropy measure adapted from its frequentist counterpart, for distinguishing between gradual and abrupt changes in the context of the proposed modelling framework.

Compared with related work in the literature, our proposed approach is unique in a number of ways. For example, Albers and Bringmann (2020). proposed an explicit modelling framework for detecting both gradual and abrupt changes in emotion dynamics with a time‐varying change point autoregressive (TVCP‐AR) model that integrated time‐varying AR and change point models. However, as noted by these authors, change point analysis is best suited for processes that undergo a limited number of change points. In most applications of change point analysis, researchers rarely hypothesize more than two change points (e.g., Chung & Maisto, 2006; Shaban, 1980; Shao et al., 2016). Misspecification of change points can have severe effects on inferential results and commonly used model fit indices have low power in detecting change point misspecification (Ning & Luo, 2017). In situations where identifying change points and corresponding individual differences is computationally expensive—such as when structural changes occur frequently (e.g. more than twice) and/or probabilistically across individuals (e.g. only some individuals exhibit changes) and over time (e.g. the timing of changes depends partly, but not deterministically, on time‐varying covariates)—RS models offer an explicit framework for representing and testing alternative scenarios of how such probabilistic and other gradual changes unfold. Another novel contribution of the present article lies in the adaptation and evaluation of a frequentist entropy measure (Asparouhov & Muthén, 2014; Celeux & Soromenho, 1996; Henson et al., 2007; Ma & Wang, 2004) to a Bayesian setting to enable model selection involving both gradual and abrupt changes and includes comparisons with information criteria measures such as the Akaike information criterion (AIC; Akaike, 1998) and Bayesian information criterion (BIC; Schwarz, 1978). Although IC measures have previously been widely used as model selection measures in growth mixture (Grimm et al., 2010) and related RS models (Chow et al., 2015, 2020), they have been found in previous research to overextract the number of mixture components in mixture models compared to entropy (Celeux & Soromenho, 1996), and little is currently known about the performance of these model selection measures when applied to models that capture both gradual and abrupt changes.

The rest of this article is organized as follows. We first present the motivating data example to illustrate possible structural changes in psychological dynamics. Then we introduce our proposed modelling framework for inspecting both gradual and abrupt changes in TVPs as well as individual differences in these changes, where we introduce several diagnostic measures that can potentially be helpful in selecting the most appropriate models to capture time‐varying changes in TVPs. Linkages between the proposed framework and other existing methods are highlighted. The performance of the proposed approaches is then evaluated through a simulation study and illustrated using an empirical example. Recommendations for model selections are provided based on both simulation and empirical results. Finally, limitations to the current work and future directions are discussed.

## 2 MOTIVATING EXAMPLE

The proposed approach was motivated by an ecological momentary intervention study (EMI; Heron & Smyth, 2010) that aimed at improving psychological well‐being (PWB) in a college‐attending, young adult population. PWB was measured through several ‘momentary’ self‐reports (up to six times a day), via surveys completed on the participants own smartphones. All participants provided informed consent prior to participation in the study. The study was designed as a 56‐day randomized controlled trial in which 160 participants were randomly assigned to three study groups: (G1) an active control group (*N* = 55) with momentary PWB surveys and no intervention; (G2) experimental group 1 (*N* = 51 after excluding four people who dropped out of the study) with momentary PWB surveys and positive practice intervention (PPI); and (G3) experimental group 2 (*N* = 54 after excluding one person who dropped out of the study) with momentary PWB surveys, positive practice and meditation intervention (PPI + Med).

The study had four main periods: (P1) pre‐intervention period (14 days); (P2) intervention period (15 days), during which G2 received PPI, G3 received PPI + Med, and G1 performed working‐memory tasks every day; (P3) post‐intervention period I (13 days); and (P4) post‐intervention period II (14 days). Each participant provided momentary assessments at irregularly spaced time intervals, up to six times a day. We aggregated the measurements to four blocks (i.e. 0–6 am, 6 am–12 pm, 12 pm–6 pm, 6 pm–12 am) to produce equally spaced data for the discrete‐time models considered in this study.

The momentary assessments of PWB were based on Seligman's PERMA model (Seligman, 2012) which consists of five dimensions of PWB—positive emotions, engagement, relationship, meaning and accomplishment. Previous research in adult populations has found that improvement in emotion was related to directional changes in dynamic characteristics, such as a gradual increase in baseline emotion and emotion regulation ability over time (Kuppens et al., 2010; Röcke & Brose, 2013; Segrin & Taylor, 2007). Furthermore, it would be reasonable to assume that the PWB dynamics of individuals can show frequent switches between distinctive stages that are characterized by unique patterns. For example, individuals with high levels of PWB can transition abruptly to a low PWB stage and vice versa. These relatively frequent progression through and reversal in change stages often occur at unknown time points (transition points).

**FIGURE 1:**
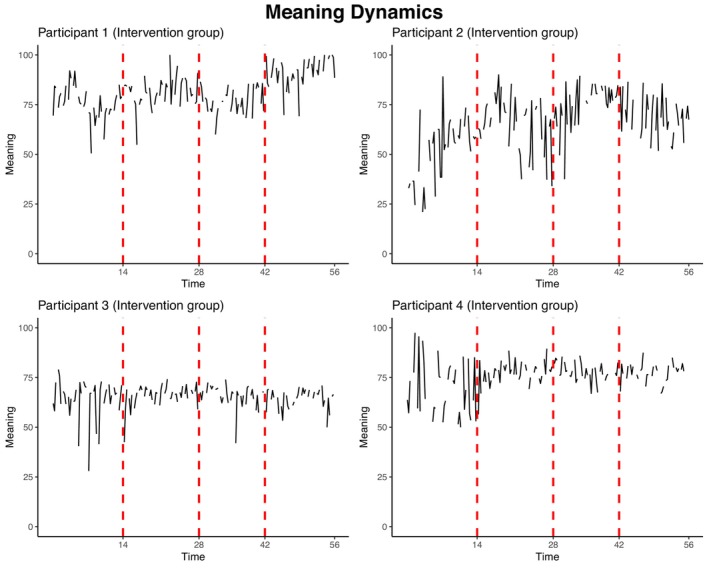
Meaning of life (MOL) dynamics of four randomly selected participants in the intervention group. The dashed lines divided the study into four periods: pre‐intervention period (14 days), intervention period (15 days), post‐intervention period I (13 days) and post‐intervention period II (14 days).

Figure 1 displays the PWB dynamics of four randomly selected participants in the study described above. For illustration purposes, we focus on the dimension of meaning of life (MOL), that captures how much an individual has a sense of purpose in life, feels that life is valuable and connects to something greater than themselves (e.g., a religious faith, a charity or a personally meaningful goal; Butler & Kern, 2016). The dashed lines in Figure 1 divided the study into four periods, P1–P4, as described above. We can see substantial individual differences in terms of how MOL levels changed over the course of the study. For example, both participants 1 and 2 experienced a gradual increase in the mean or baseline level over the course of the study, suggesting a positive intervention effect. Participants 3 and 4 showed changes not at the baseline level, but as reduced variance (i.e. smaller fluctuations) near the start of the intervention period (P1, near day 14). Compared with the change in the baseline level, the change in the variance level appeared to be more abrupt, thus motivating our representation of these abrupt changes as transitions between regimes with different variance levels in MOL.

## 3 MODEL DESCRIPTIONS

The proposed modelling framework consists of a multilevel AR model with person‐ and time‐specific changes in selected substantively motivated parameters. RS equations are used to represent how individuals manifest abrupt changes in these parameters, whereas growth curve models (GCMs) are used to depict plausible gradual changes in them. In addition, parameters that are expected to be person‐specific but time‐invariant are incorporated into the level‐2 portion of the proposed model. In the following, we describe the proposed multilevel AR model with integrated gradual and regime‐related changes in more detail.

### 3.1 Multilevel AR model

The proposed multilevel AR model is specified as:

(1) $$\begin{array}{lllllll} & \text{Level 1:}\;\; & Y_{i,t} & =\mu_{i,t}+\phi_{i}(Y_{i,t-1}-\mu_{i,t-1})+\epsilon_{i,t},\;\; & \epsilon_{i,t} & ∼N(0,{\text{IIV}}_{i,t}),\; & \end{array}$$

(2) $$\begin{array}{lllllll} & \text{Level 2:}\;\; & \phi_{i} & =\phi_{0}+\zeta_{\phi ,i},\;\; & \zeta_{\phi ,i} & ∼N(0,\sigma_{\phi }^{2}),\; & \end{array}$$

where $Y_{i,t}$ represented the value of the observed variable (e.g. MOL in the motivating example) for person i at time t. The intercept/baseline parameter, $\mu_{i,t}$, represents the baseline level around which $Y_{i,t}$ fluctuates. As one of our key TVPs of interest, $\mu_{i,t}$was made person‐ and time‐varying to reflect changes in the baseline levels of some individuals over time, such as the gradual, upward growth in the MOL of participants 1 and 2 in Figure 1. The person‐specific AR parameter, $\phi_{i}$, is constrained to fall within the range of (−1, 1) to dictate that the fluctuations around $\mu_{i,t}$, as distinct from other TVPs, are stationary, namely show invariant statistical properties across time. This is a typical constraint that has been applied to AR processes to ensure that they do not exhibit unrealistic (e.g. exploding) dynamics, and may be distinguished from other non‐stationary changes, such as growth in the mean or baseline level that occurs through $\mu_{i,t}$. In our motivating example, a higher positive $\phi_{i}$ indicates slower return to the baseline following a change caused by external events. This in turn means that the process exhibits a higher level of *inertia* or a lower level of *regulation*.

Finally, the process noise, $\epsilon_{i,t}$, followed a normal distribution with zero mean and potentially person‐ and/or time‐varying variance, typically termed intra‐individual variability (IIV) in previous studies utilizing similar modelling variations. In cases with person‐ and time‐invariant IIV, we denote that as ${\text{IIV}}_{i,t}={\text{IIV}}_{0}$. IIV was our second TVP of interest in the motivating example. A higher IIV would yield greater amplitudes of fluctuations in the process noise. Of note, we included process noise, but not measurement errors in Equation (1). As such, the IIV term may also include variance due to measurement errors (Oh et al., 2025). To maintain consistency with terminology commonly adopted in the literature, we still refer to the IIV herein as the process noise variance. In the following, we expand on extensions of the level‐1 model to incorporate gradual and abrupt changes to key modelling parameters of interest.

### 3.2 Gradual changes induced by GCMs

Inspection of the plots in Figure 1 and results from previous publications utilizing the present empirical data set (Li et al., 2024) already revealed period‐to‐period changes in the dynamic features of the participants' PWB dynamics as linked to P1–P4. Therefore, we considered the following variation of GCM to mimic the experimental design in the motivating example as well as findings in previous empirical studies:

(3) $$\begin{array}{lllll} & \mu_{i,t}=\beta_{0,i}+\beta_{1,i}{\text{Period}}_{i,p},\;\; & \ln({\text{IIV}}_{i,t})=\beta_{\text{IIV},0i}+\beta_{\text{IIV},1i}{\text{Period}}_{i,p}, & \; & \end{array}$$

(4) $$\begin{array}{lllll} & \beta_{0,i}∼N(\beta_{00},\sigma_{\beta_{0}}^{2})\;\; & \beta_{\text{IIV},0i}∼N(\beta_{\text{IIV},00},\sigma_{\beta_{\text{IIV}0}}^{2}) & \; & \\ & \beta_{1,i}∼N(\beta_{10},\sigma_{\beta_{1}}^{2})\;\; & \beta_{\text{IIV},1i}∼N(\beta_{\text{IIV},10},\sigma_{\beta_{\text{IIV}1}}^{2}) & \; & \end{array}$$

Variable ${\text{Period}}_{i,p}$ represents the *p*th (p=1,…,4) period for person i, corresponding to the four periods of the study. Under this model specification, the person‐specific intercepts, $\beta_{0,i}$, represented person i's initial level of $\mu_{i,t}$ in the first period (i.e. pre‐intervention), and the slope parameter, $\beta_{1_{i}}$, represented the change in $\mu_{i,t}$ for each distinct study period compared to the previous period. Similarly, $\beta_{\text{IIV},0i}$, and $\beta_{\text{IIV},1i}$ represent person i's pre‐intervention and across‐period slope in IIV.

### 3.3 Abrupt changes induced by RS models

To capture possible abrupt shifts in modelling parameters such as $\mu_{i,t}$ and ${\text{IIV}}_{i,t}$, we allowed them to assume different values as contingent on the latent regime of person i at time t, denoted as $S_{i,t}$:

(5) $$\begin{array}{ll} & \mu_{i,t}=\beta_{0,i}+\Delta_{\mu ,S_{i,t}}+\zeta_{i,\Delta_{\mu }}\ln({\text{IIV}}_{i,t})=\beta_{\text{IIV},0}+\Delta_{\text{IIV},S_{i,t}}+\zeta_{i,\Delta_{\text{IIV}}}\\ & \beta_{0,i}∼N(\beta_{00},\sigma_{\beta_{0}}^{2}),\zeta_{i,\Delta_{\mu }}∼N(0,\sigma_{\Delta_{\mu ,S_{i,t}}}^{2}),\zeta_{i,\Delta_{\text{IIV}}}∼N(0,\sigma_{\Delta_{\text{IIV},S_{i,t}}}^{2})\; \end{array}$$

Here, $S_{i,t}$ is an unknown person‐ and time‐specific regime indicator that can take on as many unique values as the number of hypothesized regimes, S (e.g. for a two‐regime model, S=2, $S_{i,t}\in 1,2$), whose values vary across individuals and time as:

(6) $$\begin{array}{l} P(S_{i,t}=r|S_{i,t-1}=s)=\frac{\exp(\alpha_{rs,0}+\alpha_{rs,1}{\mathrm{Period2}}_{i,t}\times {\text{Treatment}}_{i})}{\sum_{r=1}^{S}\exp(\alpha_{rs,0}+\alpha_{rs,1}{\mathrm{Period2}}_{i,t}\times {\text{Treatment}}_{i})} \end{array}$$

where r and s were indices for the regime at time t and t−1; $\Delta_{\mu_{S_{i,t}}}$ and $\Delta_{{\text{IIV}}_{S_{i,t}}}$ denote deviations in the values of baseline and IIV due to being in a specific regime, whereas $\zeta_{i,\Delta_{\mu }}$ and $\zeta_{i,\Delta_{\text{IIV}}}$ represent person i's random effects in abrupt changes in baseline and IIV, respectively, in transitioning to a new regime. These random effects are assumed to have regime‐specific variances. To facilitate interpretations of the abrupt change parameters as changes in comparison to regime 1, we set $\Delta_{\mu_{S_{i,t}}}=\Delta_{{\text{IIV}}_{S_{i,t}}}=\sigma_{\Delta_{\mu ,S_{i,t}}}^{2}=\sigma_{\Delta_{\text{IIV},S_{i,t}}}^{2}=0$ for the first regime ($S_{i,t}=1$) so that any regime‐related deviations in baseline and IIV are defined in relation to the individual's baseline MOL and sample IIV during the pre‐intervention period ($\beta_{0,i}$ and $\beta_{\text{IIV},0}$, respectively). Further constraints may be necessary, as dictated by the modelling context of interest. For example, in one of the two‐regime model motivated by the empirical study, we set $\Delta_{\mu_{S_{i,t}}}$ to be positive based on our expectation for abrupt *increases* in individuals' baseline MOL over time, due possibly to intervention or other placebo effects. Extensions to a higher number of regimes (e.g. S≥3) are also possible provided that there are sufficient information, time points and sample size to identify the differences across regimes. In situations where some study participants only experience a subset of the S hypothesized regimes postulated for the entire sample, one would still specify S to be the highest expected number of regimes. In this case, the estimated probabilities of transitioning into some of the regimes would just be relatively low for participants who did not experience all of the regimes.

The log‐odds of transitioning from regime s into regime r are assumed in Equation (6) to be a function of an intercept and a covariate. The covariate in our motivating example was an interaction term between “Period2” (0 = other periods; 1 = the intervention period, P2) and “Treatment” (0 = control group, G1; 1 = treatment group, G3), included to test possible difference between the control and treatment groups in terms of their regime‐switching patterns during the intervention period, where the effects of any group differences were expected to be the most salient relative to other periods. For example, when $\Delta_{\text{IIV},S_{i,t}}>0$ and $\Delta_{\mu ,S_{i,t}}=0$ for $S_{i,t}=2$, a positive $\alpha_{21,1}$ suggests that the treatment group is more likely to switch to a high‐volatility regime during the intervention period compared with the control group. We did not include main effects of Treatment and Period2 in Equation (6) because we did not expect systematic differences between the control and intervention group at pre‐intervention (when Period2_*i*,*t*_ = 0) due to the randomly assigned group membership or any systematic changes in MOL in the control group (for whom Treatment_*i*_ = 0) during the intervention period. For identification purposes, one of the two terms in the denominator of Equation (5) had to be designated as the reference level. Specifically, in the present study, we set staying in the same regime as the reference level by fixing $\alpha_{11,0}$, $\alpha_{11,1}$, $\alpha_{22,0}$ and $\alpha_{22,1}$ to 0 given that exploring determinants that helped predict transitions between regimes was of more interest to us.

### 3.4 Linkages between the proposed framework and existing methods

Based on the above model descriptions, we can see that the time‐varying changes in TVPs are captured by two types of models—GCM and RS models, where GCM is regarded as a type of TVP models. Below we briefly reviewed the TVP and RS models as well as their applications.

#### 3.4.1 TVP model as a representation of gradual changes

TVP models allow parameters to show variations over time or as a function of other known variables (Del Negro & Otrok, 2008; Hastie & Tibshirani, 1993; Tan et al., 2012). The TVP may be hypothesized to assume a parametric form of how it varies (e.g. linearly, quadratically) with time and other associated predictors (e.g. intervention periods, as adopted in the proposed model) or nonparametrically using relatively flexible functions, such as splines (Brumback & Rice, 1998; McKeown & Sneddon, 2014; Tan et al., 2012) and kernel methods (Hoover et al., 1998). The mixed effects generalized additive model (GAM), a semiparametric alternative that has been utilized to represent TVP (Bringmann et al., 2017; Chow et al., 2017; Wood, 2017), allows the inclusion of both parametric and nonparametric components, and for the latter, uses penalized splines to shrink unimportant coefficients and simplify model structures.

Although the proposed model assumed a parametric form with periods as predictors, it may be worth exploring other candidate models that impose less strong assumptions on the change patterns. In this article, we considered a random walk (RW) model, a semiparametric model that is popular in the state‐space TVP literature (Chow et al., 2011; Molenaar et al., 2009) to capitalize on this approach's strength at capturing more diverse person‐specific TVP trajectories than the penalized group trajectory emphasized by the GAM framework. This model is a special case of the generalized random walk (GRW) model (Gates et al., 2023; Peter & Jakeman, 1980) and is expressed as:

(7) $$\begin{array}{l} \mu_{i,t}=\mu_{i,t-1}+e_{i,t},e_{i,t}∼N(0,\sigma_{e}^{2}). \end{array}$$

The baseline, $\mu_{i,t}$, is modelled as a latent variable whose values change over time and thus has a process noise component, $e_{i,t}$. At each time step of the RW process, the TVP undergoes a random change in its value, which can be a small change or a large jump in the process, depending on the value of $e_{i,t}$. Therefore, the RW model provides a relatively flexible representation of TVPs. It reduces to a time‐invariant model when the process noise variance, $\sigma_{e}$, is equal to zero. In contrast, if the estimate of $\sigma_{e}$ is statistically greater than 0, it indicates that there is enough variability in the parameter to warrant representation as a TVP.

#### 3.4.2 RS model as a proxy for abrupt changes

With RS models, portions of individuals' data characterized by distinct patterns of means, variance‐covariance structures or other characteristics may be referred to as *regimes*. When the timing of transitioning between regimes is unknown but estimable, the resultant regimes are latent. RS models quantify the probability of transitioning between regimes by introducing such latent regime indicators and modelling the transitions between these regimes. In this way, the switches between regimes are *probabilistic* instead of deterministic—that is, we are interested in the probability of switching from one regime to the other at each time point. RS models have been applied to capture regime transitions in various fields. For instance, Neale et al. (2016) extended a latent GCM with RS to more advanced RS models and illustrated their applications using alcohol use data observed on four occasions. Others have utilized RS models to capture phases of alcohol use as interspersed with periods of abstinence (Chow et al., 2015), phases reflecting qualitatively distinct affective regulation patterns (Chow & Zhang, 2013; Lu et al., 2019) and mother‐infant movement dynamics (Chow et al., 2018).

### 3.5 Measures for detecting gradual and abrupt changes

#### 3.5.1 Uncertainty intervals

Starting with a set of parametric/non‐parametric TVP models as a possible representation for gradual changes, the presence of substantial gradual changes can be verified by examining the confidence intervals of parameters related to gradual changes (Gates et al., 2023). In our proposed model, this was accomplished by specifying an RW model for each potentially time‐varying parameter, and evaluating the process noise variance parameter (see Equation 7) as well as its corresponding uncertainty. A process noise variance that is estimated to be substantially different enough from zero indicates that the parameter shows sufficient over‐time variations to warrant representation as a TVP. In the present Bayesian framework, we obtain uncertainty estimates using summary statistics of the posterior distributions of the process noise variances, such as credible intervals (CIs), and the Highest Density Interval (HDI), a specific type of CI defined by points in regions with the highest probability density values. Thus, compared with a CI obtained from the 2.5% and 97.5% percentiles of a posterior distribution, the HDI is more effective at conveying the uncertainty associated with multi‐modal or asymmetric distributions.

The process noise variances for TVPs, just like any other variance parameters, are required to be positive to ensure valid probability distributions and stability of the Markov chain Monte Carlo (MCMC) sampling procedures. In cases where the lower bound of a HDI is close to 0, we propose to specify a Region of Practical Equivalence (ROPE) around zero to quantify the range of parameter values that are practically equivalent to zero (Kruschke, 2014). In this article, we specify the ROPE as (0, .05) for the process noise standard deviation parameter. Thus, when a HDI lower bound falls within the ROPE, we would conclude that the corresponding parameter shows insufficient evidence to be time‐varying.

Similar to the detection of gradual changes, we can detect abrupt changes by examining the estimates, CIs/HDIs in relation to the ROPE of parameters related to abrupt changes. For example, if an abrupt change in the form of intercept/baseline shifts is hypothesized, a deviation parameter representing the difference between two regimes' intercept values (e.g. $\Delta_{\mu ,S_{i,t}}$ in Equation 6), and its associated uncertainty intervals can be estimated and evaluated for evidence of abrupt shifts in the intercept/baseline level.

#### 3.5.2 IC measures

We can also regard the diagnosis of the presence of structural changes as a model selection issue. We consider three commonly used model selection metrics—the AIC, BIC and sample‐size adjusted BIC (sBIC; Sclove, 1987), all of which are based on the log‐likelihood function as:

(8) $$\begin{array}{ll} \text{AIC} & =-2\ln(\hat{L})+2k\; \end{array}$$

(9) $$\begin{array}{ll} \text{BIC} & =-2\ln(\hat{L})+k\ln(n)\; \end{array}$$

(10) $$\begin{array}{ll} \text{sBIC} & =-2\ln(\hat{L})+k\ln(\frac{n(n+2)}{24})\; \end{array}$$

where $\ln(\hat{L})$ represents the maximum of the log‐likelihood function. Here, $\hat{L}=P(Y;\hat{\theta })$ where $\hat{\theta }$ is a vector of parameter values that maximize the likelihood function, L. The number of model parameters is denoted as k and the number of total time points is denoted as $n=\sum_{i=1}^{N}T_{i}$ where $T_{i}$ is the number of time points for individual i (i=1,…,N). These IC measures impose different levels of penalty for model complexity while selecting models that produce a high log‐likelihood value. As identical to their frequentist counterparts, the AIC as shown in Equation (8) tend to favour more complex models. The BIC adds more penalty to model complexity and may prefer over‐simplified model under small sample sizes, with the sBIC proposed as a compromise between the AIC and the BIC under finite sample sizes.

Following the commonly used rule of thumb proposed by Burnham and Anderson (2004), an IC difference between two models of larger than 2 would be regarded practically significant. As an example, if the IC measures of the TVP model (e.g. RW model) is more than 2 units smaller than that of the model with time‐invariant parameters, there is some evidence favouring the presence of structural changes in TVPs (an IC difference of 10 or more would be regarded as strong evidence favourable to the TVP model).

#### 3.5.3 Entropy based on posterior regime probabilities

Motivated by the usefulness of entropy measures in the mixture modelling literature to enable selection of the number of classes/mixture components with clear separation, we propose to use entropy in the context of RS models as an additional model selection index. We adopt the entropy definition used in Mplus (Asparouhov & Muthén, 2014) as:

(11) $$E=1+\frac{1}{n\ln(K)}\overset{N}{\underset{i=1}{\sum }}\overset{T_{i}}{\underset{t=1}{\sum }}\overset{K}{\underset{k=1}{\sum }}p_{itk}\ln(p_{itk}),$$

where $p_{itk}$ represents the probability of being in the *k*th regime (k=1,…,K) for person i (i=1,…,N) at time point t ($t=1,\text{…},T_{i}$). In the Bayesian framework, an estimate for $p_{itk}$ for each person and time point is obtained by computing the proportion of posterior samples with a regime indicator value, $S_{i,t}$ of k among all posterior samples for person i and time t.

The entropy value ranges between 0 and 1, with a higher entropy value indicating better classification of mixture components. Although the performance of entropy has been widely examined in the mixture modelling literature, to the best of our knowledge, it has not yet been applied and/or evaluated in the context of simultaneous presence of gradual and abrupt changes. Thus, the strengths and weaknesses of the proposed entropy measure relative to other IC measures are one of the key research questions to be addressed in the present article. Additionally, other classification‐based performance measures, such as the Area Under the Curve (AUC; see, e.g., Bradley, 1997; Hanley & McNeil, 1982), may be used as other model selection measures given that the regime estimation is essentially a classification problem.

Once the IC measures and classification measures are obtained, we suggest implementing the model selection based on the combination of these measures. Certainly, an RS model with both satisfactory classification performance and lower IC measures should be favoured over non‐RS models. However, if an RS model could not provide clear regime classifications as indicated by low entropy and AUC values, it should not be selected even if it could yield lower IC values than non‐RS models. This indicates that the current RS model may not be suitable for capturing abrupt changes in addition to gradual changes captured by the previous TVP model. The possible solutions involve further adapting the RS model by including more predictors to improve the regime classification performance or trying other types of models.

## 4 SIMULATION STUDY

The purpose of the simulation study was to answer a series of research questions (RQs) that could help guide model selection decisions in empirical scenarios in which structural changes of the forms postulated in our motivating example are of interest to the researchers. The true data generating models used in our simulation studies were all special cases of the broader model shown in Equations ((1), (2), (3), (4), (5), (6)). All of the candidate models considered are summarized in Table 1. The RQs included:

**TABLE 1:** Models considered in the simulation study.

Models^a^	Simulation study^b^	$\mu_{i,t}$	$\ln({\text{IIV}}_{i,t})$
GCM	2a	$(\beta_{0,i}+\beta_{1}{\text{Period}}_{i,p})+e_{i,t}$	$\ln({\text{IIV}}_{0})$
RW	2a	$\mu_{i,t-1}+e_{i,t}$	$\ln({\text{IIV}}_{0})$
RW‐RS2	2a	$\left(\beta_{0,i}+\Delta_{\mu ,S_{i,t}}+\zeta_{i,\Delta_{\mu }})+(\mu_{i,t-1}+e_{i,t}\right)$; $S_{i,t}\in \{1,2\}$	$\ln({\text{IIV}}_{0})$
GCM‐RS2	1, 2a, and 3	$\left(\beta_{0,i}+\beta_{1}{\text{Period}}_{i,p})+(\Delta_{\mu ,S_{i,t}}+\zeta_{i,\Delta_{\mu }}\right)$; $S_{i,t}\in \{1,2\}$	$\ln({\text{IIV}}_{0})$
GCM + RS2‐IIV	2b	$\beta_{0,i}+\beta_{1i}{\text{Period}}_{i,p}$	$\beta_{\text{IIV},0}+\Delta_{\text{IIV},S_{i,t}}+\zeta_{i,\Delta_{\text{IIV}}}$; $S_{i,t}\in \{1,2\}$
GCM + GCM‐IIV	2b	$\beta_{0,i}+\beta_{1i}{\text{Period}}_{i,p}$	$\beta_{\text{IIV},0i}+\beta_{\text{IIV},1i}{\text{Period}}_{i,p}$
GCM‐RS3	2a	Same as GCM‐RS2 but $S_{i,t}\in \{1,2,3\}$	$\ln({\text{IIV}}_{0})$
RW‐RS3	2a	Same as RW‐RS2 but $S_{i,t}\in \{1,2,3\}$	$\ln({\text{IIV}}_{0})$

Abbreviations: GCM, growth curve model; RS2, regime‐switching models with two regimes; RS3, regime‐switching models with three regimes; RW, random walk model.

^a^ In general, we use the “+” sign in our model names to separate descriptions for $\mu_{i,t}$ (appearing prior to the “+” sign) and ${\text{IIV}}_{i,t}$ (appearing after the “+” sign). For example, “GCM + RS2‐IIV” denotes a model with GCM for $\mu_{i,t}$, but two regimes for the IIV parameter.

^b^ The column “Simulation study” marks the simulation conditions designed to address RQs 1–3, including: Simulation 1—Frequent transitions in baseline only with two sample size configurations; Simulation 2a—Frequent transitions in baseline only with *N* = 50 and *T* = 100; Simulation 2b—RS in IIV only with moderate and high sparsity with *N* = 100 and *T* = 200; Simulation 3—Frequent transitions in baseline only with different effect sizes and *N* = 50 and *T* = 100.

(Simulation 1 RQ): **How many time points are sufficient to detect both gradual changes and regime transitions in the context of the proposed model (i.e. Equations** **(1)**, **(2)**, **(3)**, **(4)**, **(5)**, **(6)** **)?** Overly frequent regime switches, particularly in the presence of a small number of time points, do not provide sufficient data to adequately identify the parameters within each regime (Chow et al., 2013). This issue is further complicated when gradual changes also exist in other dynamic features in the system. Our first RQ was aimed at providing insights on the number of time points and sample size configuration needed to identify the structural changes posited in the proposed model.

(Simulation 2 RQ): **To what extent are the diagnostic measures considered helpful for detecting different types of structural changes?** We were interested in identifying diagnostic measures that can help detect the presence of gradual and abrupt changes, the magnitudes of these changes, the correct number of regimes (if there are regime transitions), as well as individual differences in these changes. To address this RQ, we devised a range of candidate models assuming different combinations of gradual and abrupt changes on the baseline and IIV parameters and examined the extent to which the true data generation model was preferred over other candidate models under these diagnostic approaches.

(Simulation 3 RQ): **To what extent are the proposed approaches sensitive to the effect size of abrupt changes?** Gradual and abrupt changes are not entirely mutually exclusive. Distinguishing between these two types of changes have implications on the “best” actions or strategies suited for promoting desirable outcomes. However, each type of these changes can also be reasonably approximated using tools targeted for the other type of change. For instance, abrupt changes in level may be reasonably approximated using a simple TVP model without the RS structure even though larger approximation errors might be present immediately around the point of transition. The extent to which a RS model may be preferred compared to a non‐RS model representation depends in this case on the magnitude of the sudden level shifts or in other words, effect size of the abrupt change. In this simulation study, we defined an effect size measure for the overall abrupt changes in baseline levels, and evaluated how the diagnostic measures considered performed under different effect size conditions.

### 4.1 Simulation design

#### 4.1.1 Simulation Study 1

We considered two sample size conditions—the first condition set N = 100 persons and T = 200 time points to mirror the number of participants and the time series length in the empirical study. The second condition involved a smaller number of persons and time points set as N = 50 persons and T = 100 time points, designed to study whether the structural changes in the hypothesized models could be detected with less data (i.e. a smaller number of subjects and shorter time series).

A model denoted as “GCM‐RS2” was used as the true data generating model. This model included a GCM to yield gradual over‐period changes in baseline and a two‐regime model (RS2) to generate abrupt shifts in baseline due to being in regime 2 (see Table 1). Compared to the broader model in Equations ((1), (2), (3), (4), (5), (6)), we set $\ln({\text{IIV}}_{i,t})={\text{IIV}}_{0}$ to yield a person‐ and time‐invariant process noise variance. In other words, $\mu_{i,t}$ was the only truly time‐varying TVP in Simulation Study 1.

The true parameter values were set as follows. In the level‐1 AR model (see Equation 1), the AR parameter, $\phi_{i}$, was set to be a constant ϕ=.3 (i.e. no random effects) based on the results from fitting AR‐type models in previous studies (e.g., Li et al., 2022; You et al., 2019); the process noise standard deviation, $\sqrt{{\text{IIV}}_{i,t}}$, was set to a constant ψ=.5. For the GCM component, the average initial baseline was set to $\beta_{00}=0$, with corresponding random effect standard deviation of $\sigma_{\beta_{0}}=.2$. The over‐period slope was set to $\beta_{10}=.5$, with no interindividual differences in slope (i.e. $\sigma_{\beta_{1}}=0$) to allow the key source of between‐person differences in over‐time change to stem from abrupt shifts.

We defined a two‐regime special case of the RS equations in Equations (5) and (6). We set $\Delta_{\mu ,2}=2.0$ for the amount of average abrupt shift in baseline while in regime 2 as compared to regime 1 and $\sigma_{\Delta_{\mu ,2}}=.2$. As predictors of the RS log‐odds to be used in the RS equation (see Equation 6), we generated the covariate, Treatment_*i*_, as consisting of an even split of 0s and 1s, and Period2_*i*,*t*_ as a binary variable of length *T* with 0s in the first half and 1s in the second half. Aside from the coefficients that were set to 0 for identification purposes as noted earlier, $\alpha_{21,0}$ and $\alpha_{12,0}$ were set to −.5. Thus, the probabilities of transitioning between regimes when Period2_*i*,*t*_ = 0 were $\frac{\exp(-.5)}{\exp(0)+\exp(-.5)}=.38$ both from regime 1→2 and from 2→1. In contrast, the probabilities of staying within regime 1 and regime 2 were both 1−.38=.62. The parameter $\alpha_{21,1}$ was set to 1, meaning that the treatment group was more likely to switch to the high‐level regime during the intervention period (with a transition probability of .62 when Period2_*i*,*t*_ = 1 compared to .38 when Period2_*i*,*t*_ = 0). Similarly, $\alpha_{12,1}$ was set to −1, meaning that the treatment group was less likely to switch to the low‐level regime during the intervention period (with transition probability of .18 compared to .38). Compared to values of RS parameters used in prior studies (Chow et al., 2013, 2018), this specific set of RS probabilities yielded relatively frequent transitions between regime. Thus, Simulation 1 reflected a condition with *Frequent Transitions in Baseline Only* under two sample size configurations.

#### 4.1.2 Simulation Study 2

Leveraging results from Simulation Study 1, we conducted Simulation Studies 2a and 2b, respectively, with two distinct data generating models (see Table 1).

##### Simulation Study 2a

To evaluate the efficacy of the diagnostic measures considered for model selection purposes, we used the same data generating model from Simulation 1, GCM‐RS2, to generate data where $\mu_{i,t}$ was the sole TVP. We considered the following candidate models for Simulation Study 2a: the GCM, RW and RW‐RS2 (obtained by replacing the GCM component in the GCM‐RS2 with the RW), GCM‐RS2 (the true data generating model), and three‐regime extensions of GCM‐RS2 and RW‐RS2, referred to, respectively, as GCM‐RS3 and RW‐RS3 (see Table 1). The models GCM‐RS3 and RW‐RS3 served as candidate models for evaluating the performance of the fit measures in detecting the correct number of regimes.

##### Simulation Study 2b

Simulation Study 2b was motivated by visual inspection of the participants' empirical trajectories (see Figure 4), which provided some evidence for changes in IIV in some participants during and following the intervention period. Taking into consideration that shifts in variance typically require longer stretches of data to become salient than shifts in the baseline or mean, we focused on examining the effects of two RS settings under targeted sources of abrupt changes in ${\text{IIV}}_{i,t}$ with one sample size configuration (N=100 and T=200).

We considered the true data generating model, GCM + RS2‐IIV, in which we allowed $\mu_{i,t}$ to follow a cross‐period GCM with person‐specific intercept and slope; and the IIV to take on one of two possible values, depending on whether an individual was in a constant‐IIV regime 1 ($\ln({\text{IIV}}_{i,t})=\beta_{\text{IIV},0}$, where $\beta_{\text{IIV},0}$ was set to 0 to yield a IIV value of exp(0)=1 in data generation) or regime 2 with overall shift $\Delta_{\text{IIV},2}=2$ relative to $\beta_{\text{IIV},0}$ and additional person‐specific random effect, $\zeta_{i,\Delta_{\text{IIV}}}∼N(0,\sigma_{\Delta_{\text{IIV},2}}^{2})$, where $\sigma_{\Delta_{\text{IIV},2}}^{2}=.04$. The AR parameter, $\phi_{i}$, was set to be person‐specific with fixed effect $\phi_{0}$ = .3 and random effect variance of $\sigma_{\phi }^{2}=.01$. We compared the diagnostic measures for this model with those associated with another candidate model, GCM + GCM‐IIV, in which both $\mu_{it}$ and ${\text{IIV}}_{i,t}$ were specified to follow GCMs with person‐specific intercepts and across‐period slopes.

To mirror previous simulation studies in which differences between regimes became evident only over extended periods, two sets of RS parameters were used for data generation: (1) *Moderate Sparsity* with $\alpha_{21,0}=-3$, $\alpha_{12,0}=-2$, $\alpha_{21,1}=1$ and $\alpha_{12,1}=-1$; and (2) *High Sparsity* with $\alpha_{21,0}=-3.5$, $\alpha_{12,0}=-3.8$, $\alpha_{21,1}=1$ and $\alpha_{12,1}=-1$. Thus, in the Moderate Sparsity condition, the probabilities of staying within regimes 1 and 2—namely, the low‐ and high‐volatility regimes—were .95 and .88, respectively, for both the control and intervention groups during Period2_*i*,*t*_ = 0 (pre‐intervention); and .88 and .95 for the treatment group only under Period2_*i*,*t*_ = 1 (intervention period). In contrast, in the High Sparsity condition, the probabilities of staying within the low‐ and high‐volatility regimes were .97 and .98, respectively, for both groups during the pre‐intervention period; and .92 and .99 for the treatment group during the intervention period. Such higher staying probabilities were expected to yield longer consecutive stretches of data to delineate the characteristics of each regime and, in turn, clearer regime separation and entropy values.

To address RQ3, we implemented GCM‐RS2 as the true data generating model, but under three effect sizes for time‐varying changes in $\mu_{i,t}$. We defined an effect size measure to quantify the extent of sudden increase in baseline levels from the first to the second regime (see Equation 6) as: $d=\frac{\Delta_{\mu ,S_{i,t}=2}}{{\text{IIV}}_{0}/\sqrt{\left(1-\phi \right)}}$ (i.e. $\Delta_{\mu ,2}/.6$ based on the true values detailed earlier under Simulation Study 1). We considered three possible effect sizes: (1) large effect size ($\Delta_{\mu ,2}=2$ or d=3.3); (2) small effect size ($\Delta_{\mu ,2}=.6$ or d=1); and (3) null effect size ($\Delta_{\mu ,2}=0$ or d=0). The large effect size condition was the setting considered in Simulation Studies 1–2a. In this condition, the sudden shifts in $\mu_{it}$ were clearly distinguishable visually because the magnitude was more than 3 times that of the standard deviation of the system. This was in contrast to the small effect size condition, in which the small sudden shifts tended to be masked by fluctuations caused by process noises. Finally, in the null effect size condition, no true sudden shifts were present for $\mu_{i,t}$; all time‐varying changes in the intercept/baseline level were determined by the GCM component for $\mu_{i,t}$. The null condition served as a baseline for assessing the extent to which the fit measures misidentify gradual changes as abrupt.

**FIGURE 2:**
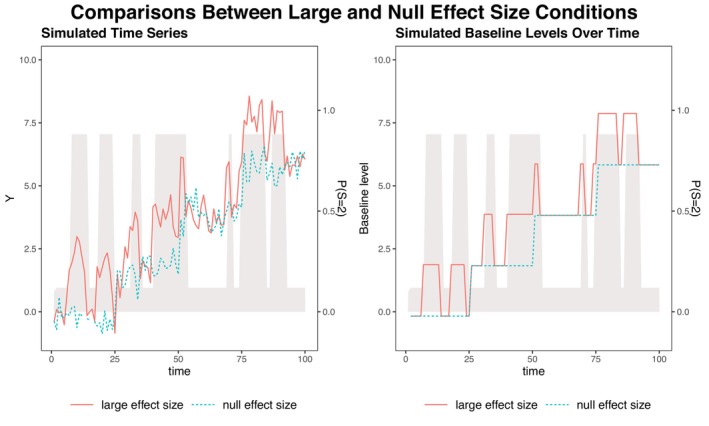
The left plot shows time series simulated based on GCM‐RS2 under the large (red solid line) and null (blue dashed line) effect size condition. The right plot shows the corresponding baseline levels (i.e. $\mu_{i,t}$) of the simulated time series in the left plot. In both plots, the right *y*‐axis represents the probability of transitioning to the high‐level regime (i.e. the height of the shaded area), whose scale is different from the left *y*‐axis.

For illustration purposes, we simulated and plotted two hypothetical time series under the large and null effect size conditions, as shown in Figure 2. From the plots, we can observe an overall growth in the baseline level under both conditions, which was determined by the GCM component. Under the large effect size condition, when the probability of transitioning to the high‐level regime (i.e. P(S=2) on the right *y*‐axis) was high, the process would exhibit a sudden increase in its baseline level (see the blue dashed line).

In summary, in the present simulation study, we generated data using two sample size configurations and three effect sizes for sudden shift in the baseline parameter. We conducted additional simulations targeting IIV as the TVP and evaluated the effects of transition sparsity on parameter recovery and model selection. For each condition, we ran 100 Monte Carlo replications. For each replication, we ran two chains, each with 20,000 iterations in total and a burn‐in of 5000 (discarded) iterations. For the additional simulation 2b targeting IIV, we collected 25,000 posterior samples per chain after a burn‐in of 5000 iterations for the GCM + GCM‐IIV model and 6000 iterations for the GCM + RS2‐IIV model, respectively. Our diagnostic criteria for MCMC sampling quality were effective sample sizes (ESSs) greater than 800 and $\hat{R}$ values below 1.05. When the true model was fitted to the simulated data, the ESS criterion was met for 99% of the parameters, with lower ESS (around 600) on variance/standard deviation parameters. In terms of convergence, convergence rates (defined as the number of replications with $\hat{R}$ below 1.05) were expected to be low when inappropriate models were fitted. Detailed convergence rates were provided in each result table below.

### 4.2 Prior specifications in simulation studies

When fitting models in the Bayesian framework, we need to assign prior distributions for unknown model parameters. The prior settings provide a principled approach for incorporating information in the analysis that is available prior to seeing the data, such as constraints on the possible ranges of the parameters. For the AR parameters, $\phi_{i}$, we imposed stationarity constraints both on the group‐level mean, $\phi_{0}$, as well as the person‐level AR parameters, $\phi_{i}$, in conditions where between‐individual differences in AR were allowed. Specifically, we assigned normal distributions that were truncated at −1 and 1, respectively, to the prior $\phi_{0}∼{𝒩}_{\left[-1,1\right]}(0,1)$ and distributional assumption of $\phi_{i}∼{𝒩}_{\left[-1,1\right]}(\phi_{0},\sigma_{\phi }^{2})$.

We used weakly informative priors, whenever plausible, on other parameters. For fixed effects parameters in the baseline level ($\mu_{i,t}$), including $\beta_{00}$ and $\beta_{10}$, we assigned normal distributions with a mean of 0 and a large variance, 𝒩(0,100). For the shift in baseline parameter in the GCM‐RS2, we assigned a uniform distribution, 𝒰(0, 10), to $\Delta_{\mu ,2}$ to enforce the expectation for a positive average shift (increase) in the participants' baseline level in the high‐level regime (regime 2). For the corresponding GCM describing gradual changes in the log IIV parameter (*ln*(IIV_*i*,*t*_)) in the GCM + GCM‐IIV model, the fixed effects for the intercept ($\beta_{\text{IIV},00}$) and slope ($\beta_{\text{IIV},10}$) were given priors of 𝒩(0,10).

For all random effect variance parameters (e.g. $\sigma_{\phi }^{2}$, $\sigma_{\beta_{0}}^{2}$, $\sigma_{\Delta_{\mu ,2}}^{2}$ and $\sigma_{\Delta_{\text{IIV},2}}^{2}$), we assigned non‐informative Inverse‐Gamma priors, ℐ𝒢(.001, .001), with a few minor exceptions. For instance, to improve ESS and speed convergence (based on $\hat{R}$ values), we assigned a uniform prior, 𝒰(0, 10), to the random‐effect standard deviation parameters for the ln(IIV) intercept and slope ($\sigma_{\beta_{\text{IIV},0}}$ and $\sigma_{\beta_{\text{IIV},1}}$); and a prior of 𝒰(0, 1) for $\sigma_{\phi }$ in the GCM + GCM‐IIV model.

The coefficients governing the log‐odds of the transition probabilities, $\alpha_{rs,k}$, were generally assigned a prior of 𝒩(0,10). For identification purposes, the log‐odds of remaining in the same regime were fixed at 0. For the RS components in the GCM + RS2‐IIV model, we assigned a uniform prior, 𝒰(0, 10), for the average shift in $\ln({\text{IIV}}_{i,t})$ for the second regime, $\Delta_{\text{IIV},2}$. The lower limit of 0 constrained the average shift in IIV to be positive in the second regime, thus dictating the second regime as a high‐volatility or ‘high‐IIV’ regime.

### 4.3 Performance measures

Several performance measures were used to summarize the results from addressing the RQs. For instance, to assess the quality of the estimates, we used the root mean square error (RMSE) to measure the average difference between estimated and actual values. In addition, we also considered *sensitivity* (similar to power in the frequentist framework), defined as the proportion of Monte Carlo replications whose HDIs did not contain 0. A higher sensitivity value indicated more evidence in detecting the effect size or high power for detecting the effect size if conceptually understood in the frequentist framework. In particular, for parameters whose true values were close to zero, we defined the sensitivity measure as the proportion of replications whose HDIs completely fell outside the ROPE (e.g. (0, .05) for the process noise standard deviation parameter). We also considered *type I error rates*, defined as the proportion of replications whose HDIs did not contain 0 when the true value was 0. A type I error rate higher than 5% indicated the false rejection of the null effect.

## 5 SIMULATION RESULTS

### 5.1 RQ1: Sample size requirements

We found that when the true model (i.e. GCM‐RS2) was fitted, all model parameters were well recovered even under the small sample size condition (N=50, T=100) (see Tables 2, 3, 4, 5). The entropy and AUC scores were relatively high, indicating that this 2‐regime model could provide clear regime classifications. More detailed elaborations on the efficacy of different diagnostic and model selection measures under this particular sample size configuration are summarized in the context of addressing RQ2.

**TABLE 2:** Results from Simulation Studies 1 and 2a. Comparisons of estimation and model fit results across models (*N* = 50, *T* = 100, large effect size).

Parameter	True values	RW	GCM	RS2‐GCM	RS2‐RW	RS3‐GCM	RS3‐RW
Parameters in the AR component							
ϕ	.3	.05	.07	.02	.03	.02	.03
ψ	.5	.58	.45	.01	.01	.01	.01
Parameters in the GCM component							
$\beta_{00}$	0	1.12	1.1	.04	.06	.04	.06
$\beta_{1}$	.5	—	.04	.01	—	.01	—
Parameters in the RS component							
$\Delta_{\mu ,2}$	2	—	—	.03	.04	.17	.11
$\alpha_{21,0}$	−.5	—	—	.04	.05	.55	.29
$\alpha_{21,1}$	1	—	—	.17	.17	—	—
$\alpha_{12,0}$	−.5	—	—	.05	.05	.21	.31
$\alpha_{12,1}$	−1	—	—	.14	.13	—	—
AIC		14,893	13,505	7319	7176	7263	7111
BIC		14,913	13,544	7391	7242	7360	7209
sBIC		14,929	13,576	7450	7295	7440	7289
Entropy		—	—	.91	.89	.85	.81
AUC		—	—	1	1	.89	.91
Sensitivity ($\sigma_{e}$)		1	.96	—	1	—	1
% of Convergence		100	50	100	100	72	72

*Note*: The upper panel displays the true values and RMSEs for the parameters obtained from different models; the lower panel displays various diagnostic measures across different models.

**TABLE 3:** Results from Simulation Study 2b. Comparisons of estimation and model fit results for moderate and high sparsity transitionsin IIV (*N* = 100, *T* = 200).

Parameter	True value(s)	Model			
		Moderate sparsity		High sparsity	
		GCM + GCM‐IIV	GCM + RS2‐IIV	GCM + GCM‐IIV	GCM + RS2‐IIV
Parameters in the $\mu_{i,t}$					
AR component					
$\phi_{0}$	.30	.30	.30	.30	.30
$\sigma_{\phi }$	.10	.11	.10	.11	.10
GCM component					
$\beta_{00}$	.00	.00	.00	.00	.00
$\sigma_{\beta_{0}}$	.20	.17	.19	.17	.19
$\beta_{10}$	.50	.50	.50	.50	.50
$\sigma_{\beta_{1}}$	.20	.20	.20	.20	.20
Parameters in the IIV					
RS component					
$\Delta_{\text{IIV},2}$	2.00	—	2.00	—	1.99
$\sigma_{\Delta_{\text{IIV},2}}$	.20	—	.20	—	.20
$\alpha_{21,0}$	−3.0 /−3.5	—	−3.00	—	−3.54
$\alpha_{12,0}$	−2.0/−3.8	—	−2.02	—	−3.86
$\alpha_{21,1}$	1.00	—	1	—	1.01
$\alpha_{12,1}$	−1.00	—	−1.01	—	−1.02
GCM component					
$\beta_{\text{IIV},0}$	—	1.12	—	1.41	—
$\sigma_{\beta_{\text{IIV},0}}$	—	.28	—	.35	—
$\beta_{\text{IIV},1}$	—	.01	—	.03	—
$\sigma_{\beta_{\text{IIV},1}}$	—	.13	—	.16	—
Model fit and diagnostics					
AIC		79,306	70,463	85,795	78,461
BIC		79,385	70,566	85,874	78,564
sBIC		79,452	70,653	85,941	78,650
Entropy		—	.58	—	.69
AUC		—	.94	—	.97
Recall		—	.76	—	.91
Precision		—	.87	—	.92

*Note*: The table presents parameter estimates and model fit diagnostics from two models fitted to simulated data (*N* = 100, *T* = 200). GCM + GCMIIV = growth curve model, which models gradual changes in both the $\mu_{i,t}$ and IIV across study periods. GCM + RS2‐IIV = a model combining the GCM for the $\mu_{i,t}$ with a two‐regime switching (RS) component for the IIV. “Moderate Sparsity” and “High Sparsity” refer to simulation conditions generated with different true parameters governing the log‐odds of transitioning between the two IIV regimes. The “Moderate Sparsity” condition used $\alpha_{21,0}$ and $\alpha_{12,0}$ values of −3.0 and −2.0, whereas the “High Sparsity” condition used more negative α values of −3.5 and −3.8, resulting in a lower probability of switching between regimes.

**TABLE 4:** Results from Simulation Study 3. Comparisons of estimation and model fit results across models (*N* = 50, *T* = 100, small effect size).

Parameter	True values	RW	GCM	RS2‐GCM	RS2‐RW	RS3‐GCM	RS3‐RW
Parameters in the AR component							
ϕ	.3	.03	.03	.02	.03	.03	.03
ψ	.5	.08	.06	.02	.02	.02	.02
Parameters in the GCM component							
$\beta_{00}$	0	.34	.33	.08	.1	.1	.13
$\beta_{1}$	.5	—	.02	.01	—	.01	—
Parameters in the RS component							
$\Delta_{\mu ,2}$	.6	—	—	.09	.11	.21	.26
$\alpha_{21,0}$	−.5	—	—	.24	.31	.46	1.3
$\alpha_{21,1}$	1	—	—	1.19	1.36	—	—
$\alpha_{12,0}$	−.5	—	—	.3	.33	.68	1.52
$\alpha_{12,1}$	−1	—	—	.93	.69	—	—
AIC		8745	8239	7547	7444	7432	7333
BIC		8764	8278	7618	7509	7530	7431
sBIC		8780	8310	7677	7563	7610	7511
Entropy		—	—	.21	.19	.37	.38
AUC		—	—	.8	.79	.78	.72
Sensitivity ($\sigma_{e}$)		1	.19	—	1	—	1
% of Convergence		100	54	78	94	36	20

*Note*: The upper panel displays the true values and RMSEs for the parameters obtained from different models; the lower panel displays various diagnostic measures across different models.

**TABLE 5:** Results from Simulation Study 3. Comparisons of estimation and model fit results across models (*N* = 50, *T* = 100, null effect size).

Parameter	True Values	RW	GCM	RS2‐GCM	RS2‐RW	RS3‐GCM	RS3‐RW
Parameters in the AR component							
ϕ	.3	.02	.02	.01	.02	.02	.02
ψ	.5	.01	.02	.01	.03	.02	.06
Parameters in the GCM component							
$\beta_{00}$	0	.05	.03	.18	.07	.16	.14
$\beta_{1}$	.5	—	.01	.01	—	.01	—
Parameters in the RS component							
$\Delta_{\mu ,2}$	0	—	—	.29	.46	.21	.25
$\alpha_{21,0}$	−.5	—	—	2.46	2.18	1.47	3.13
$\alpha_{12,0}$	−.5	—	—	3.34	1.01	1.61	1.33
AIC		7172	6910	7209	6895	7015	6395
BIC		7205	6949	7281	6967	7113	6493
sBIC		7232	6981	7339	7026	7193	6573
Entropy		—	—	.37	.22	.34	.19
AUC		—	—	.51	.51	.51	.51
Sensitivity ($\sigma_{e}$)		1	.03	—	1	—	1
% of Convergence		100	78	44	26	24	8

*Note*: The upper panel displays the true values and RMSEs for the parameters obtained from different models; the lower panel displays various diagnostic measures across different models.

### 5.2 RQ2: Efficacy of diagnostic measures in detecting the true change pattern

RQ2 sought to clarify whether the diagnostic measures (e.g. uncertainty intervals, IC measures, entropy, AUC scores) would favour the true data generation model. We begin by summarizing results from the condition with a large effect size of sudden shift in $\mu_{i,t}$ (see Table 2). Overall, the non‐RS models yielded higher RMSEs than the RS models because they failed to capture the abrupt changes in $\mu_{i,t}$. As a result, the initial baseline level, $\beta_{00}$, was more biased under RW and GCM. Also, part of the variability in $\mu_{i,t}$ that should have been captured by the RS component was captured by the AR process noise in RW and GCM, thus resulting in biased estimates of the AR process noise standard deviation (i.e. ψ). Among RS models, 3‐regime RS models yielded higher RMSEs than 2‐regime RS models in terms of parameters in the RS component, which was expected given that the true data generation model was a 2‐regime model.

In terms of the performance of multiple diagnostic measures, we first examined the uncertainty interval for the process noise standard deviation parameter (i.e. $\sigma_{e}$) to see if it successfully flagged the presence of any structural changes. The sensitivity value for $\sigma_{e}$ was close to 1 under RW, GCM and RW‐RS, which means that the HDIs were outside of the ROPE (i.e. (0, .05)) across (almost) all replications, suggesting that these models successfully detected the time‐varying changes in $\mu_{i,t}$. In particular, a sensitivity value of .96 under GCM suggested that in additional to period‐to‐period changes captured by the GCM, there were other types of time‐varying changes not captured by the GCM, which according to the simulation design were the sudden shifts.

In terms of other diagnostic measures, although 3‐regime models yielded lower IC values than their 2‐regime counterparts, both entropy and AUC scores favoured 2‐regime models, suggesting that compared to IC measures, entropy and AUC might be more reliable in selecting the true number of regimes in the presence of sudden shifts in means due to $\mu_{i,t}$. Finally, among the two 2‐regime models, although the IC measures favoured the RW‐RS2 model, the entropy measure favoured the GCM‐RS2, the true model.

Table 3 shows the results from Simulation 2b, in which the goal was to assess the efficacy of selected diagnostic measures in distinguishing abrupt from gradual changes in IIV. We found in this case that all IC measures correctly selected the true data generating model, GCM + RS2‐IIV, relative to the candidate model, GCM + GCM‐IIV. In addition, when the misspecified GCM + GCM‐IIV model was fitted, the person‐specific abrupt shifts in IIV for the treatment group during the intervention period were “absorbed” largely into person‐specific GCM intercepts and over‐period slopes for the IIV, with little influence on other remaining parameters, which were well recovered. As expected, fitting the true GCM + RS2‐IIV model to data from the High Sparsity condition was characterized by higher entropy value (.69), AUC (.97), recall (.91) and precision (.92) than those obtained in the Moderate Sparsity condition (with corresponding entropy, AUC, recall and precision values of .58, .94, .76 and .87, respectively). Despite the lower entropy values compared to conventional standards for reliable class separation in (cross‐sectional) mixture models (e.g., with an entropy >.9; Celeux & Soromenho, 1996), the point estimates and AUC from GCM + RS2‐IIV were still satisfactory with entropy values that were close to .6—both in terms of the estimated model's ability to capture meaningful between‐regime differences in intraindividual change patterns and properties of the classification results. This suggested that slightly lower thresholds may be reasonable—or even expected—for longitudinal RS models.

We found in follow‐up simulations that simultaneous freeing of the ln(IIV) intercept, $\beta_{\text{IIV},0}$ and random effect variance of person‐specific deviations in abrupt change in IIV, $\sigma_{\Delta_{\text{IIV}}}^{2}$, was a direct contributor of low entropy values. Table 3 shows the results when $\beta_{\text{IIV},0}$ was fixed to its true value of 0. When it was freely estimated, satisfactory results with entropy >.7 were observed when no interindividual differences in IIV shift were allowed (i.e. when $\sigma_{\Delta_{\text{IIV},2}}^{2}$ was set to 0). This observation helped guide our decisions to simplify the random effect structures for the empirical study by setting $\sigma_{\Delta_{\text{IIV},2}}^{2}$ to 0 and freely estimating the ln(IIV) intercept, $\beta_{\text{IIV},0}$.

Overall, we found that multiple candidate models could at times work well and even yielded lower IC measures than the true data generating models. However, under large effect size conditions, using the IC measures in combination with entropy was effective at selecting the true data generating models over other candidate models in the presence of sudden shifts in baseline or IIV values.

### 5.3 RQ3: Sensitivity to the effect size of the abrupt shift in the intercept/baseline level

This question was addressed by comparing results from simulation conditions with a null/small/large effect size in the overall abrupt change in $\mu_{i,t}$. Results are summarized in Tables 2, 3, 4, 5.

Table 4 shows the simulation results under the small effect size condition. Overall, reducing the effect size of sudden shifts impaired the parameter estimation accuracy and regime classification performance of all RS models and also reduced the discrepancy between different models in terms of their parameter estimates and model fit indices. For instance, the biases in the AR process noise standard deviation parameter and the initial baseline level parameter were not as severe as those under the large effect size condition. The sensitivity value for $\sigma_{e}$ under GCM was only .19, suggesting less evidence in detecting other types of time‐varying changes in additional to period‐to‐period changes captured by the GCM. Furthermore, the entropy scores were below .4 for all RS models, indicating poor performance in distinguishing between different regimes. In contrast to the low entropy scores, the AUC scores were still acceptable (around .8). Under this condition, including the RS component into the model seemed to be less critical given the less distinct discrepancy in IC measures between non‐RS and RS models. These performance changes were reasonable since the magnitude of the sudden shifts were comparable to the overall standard deviation of the time series under this small effect size condition, in which scenario modelling such small sudden shifts would not lead to a substantive increase in model fits. We also investigated whether the performance of RS models in classifying different regimes would be improved under the large sample size condition. Although there was a slight increase in entropy under the large sample size condition, the entropy values were still below .4.

Table 5 showed the simulation results under the null effect size condition. Under this condition, the GCM performed the best in recovering the parameters since a GCM‐RS2 with null sudden shifts was essentially a GCM. The sensitivity value for $\sigma_{e}$ under GCM was further reduced to .03, which was a strong evidence that the GCM was sufficient to capture time‐varying changes in $\mu_{i,t}$. Both entropy and AUC scores for RS models were low, thus ruling out RS models. The IC measures were less informative in model selection.

Typically, when we observed such low sensitivity value for $\sigma_{e}$ under GCM, we would stop at the non‐RS model without further trying RS models. However, under this null effect size condition, it would be interesting to examine whether there was a false detection of sudden shifts when RS models were fitted, which would involve checking the type I error rates for regime‐dependent intercept parameters (e.g. $\Delta_{\mu ,2}$). Recall that the type I error rate was defined as the proportion of replications whose HDIs did not contain 0 when the true value was 0. A type I error rate higher than 5% indicated the false rejection of the null effect (in our case, the false detection of sudden shifts). Results showed that the type I error rate for $\gamma_{0}$ was 0 when GCM‐RS2 was fitted and was .8 when RW‐RS2 was fitted, which were acceptable.

In terms of computational time, under the small sample size condition (*N* = 50, *T* = 100), it took about 15 min to run non‐RS models (e.g. RW, GCM) and 2.5 h to run RS models (e.g. RW‐RS2, GCM‐RS2). Under the large sample size condition (N=100, T=200), the computational time was about 1 h for non‐RS models and 14 h for RS models.

Overall, the above results again highlighted the utility of the entropy measure in model selection. The entropy values were instrumental in helping to determine characteristics of the RS models. In situations where the entropy values are very low or if the variability in a particular parameter is small (e.g. a relatively low sensitivity value for $\sigma_{e}$ under GCM), then a simpler model such as the GCM might be selected over other more complex models.

### 5.4 Recommendations for model selections

In this simulation study, we evaluated the performance of four types of model selection metrics—IC measures, entropy, AUC and sensitivity. To facilitate the comparison, we summarized the model selection results under different decision rules in Table 6. The decision rules considered were as follows.

- IC measures—the model with the lowest IC value was selected.
- Entropy/AUC + IC measures—among all RS models with entropy/AUC values higher than .6, the model with the highest entropy/AUC value was selected; if no RS models yielded entropy/AUC values higher than .6, then the non‐RS model with the lowest IC value was selected.
- Entropy/AUC + Sensitivity—among all RS models with entropy/AUC values higher than .6, the model with the highest entropy/AUC value was selected; if no RS models yielded entropy/AUC values higher than .6, then check if there were non‐RS models with sensitivity values lower than .2. If so, the non‐RS model with the lowest sensitivity value was selected, otherwise no model would be selected.

**TABLE 6:** Summary of model selection results across all three simulation studies based on different combinations of metrics.

Simulation study	True model	Effect size	IC measures	Entropy + IC	AUC + IC	Entropy + sensitivity	AUC + sensitivity
3	GCM‐RS2	Large	RW‐RS3	GCM‐RS2	GCM‐RS2/RW‐RS2	GCM‐RS2	GCM‐RS2/RW‐RS2
3	GCM‐RS2	Small	RW‐RS3	GCM	GCM‐RS2	GCM	GCM‐RS2
3	GCM	Null	RW‐RS3	GCM	GCM	GCM	GCM
2b	GCM + RS2‐IIV	Large	GCM + RS2‐IIV	GCM + GCM‐IIV	GCM + RS2‐IIV	Not applied	Not applied

*Note*: The true model in the last row (GCM + RS2‐IIV) corresponds to Simulation 2b and summarizes results from both the moderate and high sparsity conditions IIV. For this simulation condition, only the GCM + GCM‐IIV and GCM + RS2‐IIV models were compared.

First, IC measures failed to select the true models across all conditions. The “entropy + IC” and “entropy + sensitivity” decision rules could select the true models across all conditions except for the small effect size condition, while the “AUC + IC” and “AUC + sensitivity” decision rules could select the true models across all conditions. In fact, the small effect condition may be practically regarded as no sudden shifts since it would be hard to distinguish between sudden shifts and fluctuations caused by process noises, hence it was reasonable for the “entropy + IC” and “entropy + sensitivity” decision rules to choose non‐RS models instead of RS models under this specific condition.

Therefore, based on all the simulation results, we would conclude that the combination of entropy/AUC and IC/sensitivity measures were most effective in selecting the true model in the simulation setting. However, since the calculation of AUC scores requires knowing the true values, and sensitivity has to be obtained via simulation, which cannot be easily conducted in real world data analysis, the most practically helpful decision rule would be the combination of entropy and IC measures.

## 6 EMPIRICAL STUDY

Previous analysis of the empirical data set in the motivating example section already showed improved MOL in both control and intervention groups, as indicated by increased regulation and decreased IIV (i.e. ϕ and $\psi^{2}$ in Equation 1, respectively) levels of MOL dynamics over the four periods of the study. Such period‐to‐period changes were not found to be different between the control (G1) and intervention (G3) group, suggesting that the PPI + Med intervention might not have added benefit at the group level (Li et al., 2024). However, these previous results highlighted only the gradual changes in dynamics as related to intervention periods, but did not address the possibility of abrupt changes or regime transitions in dynamics. From Figure 1 in the motivating example section, it would be reasonable to assume abrupt changes, for instance, in the participants' baseline and IIV levels. Therefore, as an extension to the previous work, we fit a series of models with gradual and abrupt changes in the baseline and IIV parameters for the participants' MOL dynamics.

We focused on comparing participants in the control group (G1) with those in intervention group G3, who received the full intervention (i.e. both positive practices and meditation). We considered the following research questions related to MOL dynamics:

- RQ1: Were there any between‐individual differences in over‐period changes in the participants' baseline levels of MOL?
- RQ2: Were there any regime transitions in the baseline/IIV levels of MOL?
- RQ3: Was the intervention group more likely to transition to a higher‐baseline/lower‐IIV regime during the intervention period?
- RQ4: For all the models with gradual and abrupt changes considered, what model best captured the MOL dynamics of the sample as a whole?

We used the RW model as an initial exploration of the presence of TVPs, and considered two additional candidate models, GCM + RS2‐IIV and GCM + GCM‐IIV (see Table 1) to the empirical data. Both of these models allowed us to answer RQ1, whether there were substantial between‐individual differences in the across‐period growth rate, as determined by the statistical significance of the random effect variance, $\sigma_{\beta_{1}}^{2}$.

The RS portion of GCM + RS2‐IIV was as defined in Equation (6). RQ2 and RQ3 were addressed by examining model selection results and parameter estimates for targeted RS logodd parameters. To address RQ2, we compared the model selection measures for GCM + RS2‐IIV and GCM + GCM‐IIV to elucidate whether the time‐varying changes in IIV, if any, were gradual (i.e. better described by the GCM) or abrupt (i.e. better described by a RS model with $\Delta_{\text{IIV},s}$ that was credibly different from 0). To investigate whether the intervention group was more likely to transition into a specific regime during the intervention period (i.e. RQ3), we examined whether $\alpha_{rs,1}$ was credibly different from 0. Finally, for RQ4, we evaluated model selection measures such as IC and entropy values to select a preferred model among all the candidate models.

The prior specifications generally mirrored the weakly informative prior settings used in the simulation studies, except for a few adaptations. Specifically, priors for the GCM portion of the baseline level ($\mu_{i,t}$) in the GCM‐RS2 model were adjusted slightly to values that were reasonable for the data (e.g. $\beta_{00}$ was assigned a normal prior of 𝒩(50,100), $\sigma_{\beta_{00}}$ was assigned a prior of 𝒰(0, 5)), and we imposed an empirical constraint that person‐specific baseline shifts in regime 2 follow a normal distribution truncated to the interval [−8, 8], namely, ${𝒩}_{\left[-8,8\right]}(\Delta_{\mu ,2},\sigma_{\Delta_{\mu ,2}}^{2})$. We specified the time‐ and person invariant ln(IIV) in the GCM‐RS2 model and the IIV in regime 1 of the GCM + RS2‐IIV to have a prior of 𝒩(0,100). As in Simulation Studies 1 and 2a, no interindividual differences in GCM slope were allowed for $\mu_{i,t}$ in GCM‐RS2 by setting $\sigma_{\beta_{1}}^{2}$ to 0 to restrict most over‐time changes in $\mu_{i,t}$ to be captured by the abrupt shift parameters for $\mu_{i,t}$, such as $\Delta_{\mu ,2}$. In contrast, $\sigma_{\beta_{1}}$ was freely estimated and assigned a prior of 𝒰(0,5) in the GCM + RS2‐IIV model given the satisfactory recovery of this parameter in the presence of abrupt shifts in IIV in Simulation Study 2b. In addition, we assigned a 𝒰(0, 10) prior to $\Delta_{\mu ,2}$, with a relatively diffuse prior of 𝒰(0, 10) for the random effect standard deviation for the abrupt shift in baseline $\sigma_{\Delta_{\mu ,2}}$.

For the RS components in the GCM + RS2‐IIV model, we assigned a prior of U(−10,10) to $\Delta_{\text{IIV},2}$ given the lack of clear rationale for the expected direction of change in IIV while in the second regime, postulated to follow the intervention period. As distinct from Simulation 2b, we set $\sigma_{\Delta_{\text{IIV},2}}$ to 0—thus allowing for no interindividual differences in the amount of sudden shift in IIV—to more clearly distinguish between the regimes. To avoid extreme posterior samples of log‐odds that translate to negligible changes on the probability scale in both the GCM‐RS2 and GCM + RS2‐IIV models, the RS coefficients of $\alpha_{rs,k}$ were assigned a relatively informative prior of 𝒩(0,1), in contrast to the 𝒩(0,10) priors used in the simulation studies. Other priors and constraints were identical to those used in the simulation studies.

### 6.1 Empirical results

In the Bayesian model fitting procedure, we ran two chains, each with 30,000 iterations in total and a burn‐in of 6000 (discarded) iterations. We used the same criteria as for the simulation study to test MCMC sampling quality and found that $\hat{R}$ was lower than 1.05 for all parameters. The ESS was above 1500 for all parameters except for some of the RS log‐odds coefficients in the GCM‐RS2 and GCM + RS2‐IIV models. In the former, all of the RS log‐odds coefficients showed ESS of 460 or lower, whereas in the latter, only one RS log‐odds coefficient ($\alpha_{12,0}$) showed an ESS that was below 700.

We first fit an RW model to each of the three parameters (i.e. $\mu_{i,t}$, $\phi_{i,t}$ and ${\text{IIV}}_{i,t}$) and found substantial time‐varying changes in the baseline and IIV parameters, as indicated by the large process noise variance in the RW model. Based on results from the RW model, we only retained time‐varying changes in baseline and IIV levels in our subsequent models.

Results from fitting the GCM‐RS2 and GCM + RS2‐IIV models are summarized in Table 7. Of the two models, GCM + RS2‐IIV, the model postulating gradual changes in the participants' person‐specific baseline levels but abrupt shifts in IIV yielded a better fit than the GCM + RS2 model in terms of lower IC measures and higher entropy. We thus focus on elaborating results from the GCM + RS2‐IIV model. Inspection of the GCM parameter estimates associated with $\mu_{i,t}$ in the GCM + RS2‐IIV model indicated that the overall initial baseline level of MOL was 78.39, with substantial individual differences (${\hat{\sigma }}_{\beta_{0}}=4.99$). No credible gradual over‐time changes in baseline were found on average (i.e. ${\hat{\beta }}_{10}$ was not credibly different from 0), although credible between‐individual differences were found (see $\sigma_{\beta_{1}}$). In sum, to address RQ1, we found no over‐period, gradual change in the group's baseline on average, but substantial between‐participant differences in baseline were found.

**TABLE 7:** Results obtained from GCM‐RS2 and GCM + RS2‐IIV to the empirical Go‐HIAR data.

Parameter	Model			
	GCM‐RS2		GCM + RS2‐IIV	
	Estimates	95% HDI	Estimates	95% HDI
Parameters in the $\mu_{i,t}$				
AR component				
$\phi_{0}$	.48	[.43, .54]	.43	[.37, .48]
$\sigma_{\phi }$	.25	[.21, .30]	.25	[.21, .30]
GCM component				
$\beta_{00}$	73.71	[71.81, 75.66]	78.39	[77.38, 79.40]
$\sigma_{\beta_{0}}$	9.94	[9.81, 10.00]	4.99	[4.97, 5.00]
$\beta_{10}$	.29	[.10, .47]	−.37	[−1.02, .27]
$\sigma_{\beta_{1}}$	—	—	3.23	[2.78, 3.74]
RS component				
$\Delta_{\mu ,2}$	6.09	[3.47, 9.66]	—	—
$\sigma_{\Delta_{\mu ,2}}$	5.63	[3.70, 7.76]	—	—
$\alpha_{21,0}$	1.478	[1.06, 1.91]	—	—
$\alpha_{12,0}$	.02	[−.23, .28]	—	—
$\alpha_{21,1}$	.273	[−.90, 1.58]	—	—
$\alpha_{12,1}$	−.12	[−.74, .49]	—	—
Parameters in the ${\text{IIV}}_{i,t}$				
${\text{IIV}}_{0}$	46.87	[45.74, 48.08]	—	—
RS component				
$\beta_{\text{IIV},0}$	—	—	2.65	[2.61, 2.69]
$\Delta_{\text{IIV},2}$	—	—	2.58	[2.52, 2.64]
$\alpha_{21,0}$	—	—	−3.85	[−4.02, −3.67]
$\alpha_{12,0}$	—	—	−2.69	[−2.87, −2.50]
$\alpha_{21,1}$	—	—	.09	[−.31, .51]
$\alpha_{12,1}$	—	—	−.01	[−.45, .44]
Model fit and diagnostics				
AIC	109,854		101,349	
BIC	109,942		101,446	
sBIC	110,019		101,529	
Entropy	.11		.76	

*Note*: The table presents parameter estimates and model fit diagnostics from two models fitted to empirical (*N* = 108, *T* = 224). GCM‐RS2 = growth curve model, which models both gradual changes and regime switching (RS) changes in the $\mu_{i,t}$. GCM + RS2‐IIV = a model combining the GCM for the $\mu_{i,t}$ with a RS component for the IIV.

In terms of RQ2, the GCM + RS2‐IIV yielded an overall initial IIV level of $e^{2.65}=14.15$, with a credible positive deviation in IIV while in regime 2 (see ${\hat{\Delta }}_{\text{IIV},2}=2.58$ under “Parameters in the IIV”). Note that when fitting models to IIV, we log‐transformed the IIV variable (which as a variance parameter was limited to take non‐negative values) to place it on the real line for regression modelling. That is, $\Delta_{\text{IIV},2}=\ln({\text{IIV}}_{\text{regime}2})-\ln({\text{IIV}}_{\text{regime}1})$. In other words, $\frac{{\text{IIV}}_{\text{regime}2}}{{\text{IIV}}_{\text{regime}1}}=e^{\Delta_{\text{IIV},2}}$. Therefore, ${\hat{\Delta }}_{\text{IIV},2}=2.58$ indicated that the IIV level in regime 2 was 2.58 times higher than that in regime 1 (here regime 1 represented the low‐volatility regime).

To address RQ3, we found no credible difference between the control and intervention group in the transition log‐odds during the intervention period (i.e. neither ${\hat{\alpha }}_{12,1}$ nor ${\hat{\alpha }}_{21,1}$ were credibly different from 0). For RQ4, we found that GCM + RS2‐IIV model offered reasonable separation between the two identified regimes, with an entropy of .76. Supplementing our answers to other RQs, we also observed a moderate level of inertia in the MOL dynamics of participants, as indicated by an AR coefficient of .43, as well as individual differences in magnitudes of inertia, as indicated by a random‐effect standard deviation of .25, with an HDI outside of ROPE.

Compared to the GCM + RS2‐IIV, the GCM‐RS2 yielded disparate conclusions from those suggested by GCM + RS2‐IIV in the following ways. First, in GCM‐RS2, the average change over the period in $\mu_{i,t}$ (i.e. ${\hat{\beta }}_{10}$) was estimated to be positive and credibly different from 0. This was in contrast to the lack of credible average over‐period slope for $\mu_{i,t}$ as revealed by the GCM + RS2‐IIV. Second, a positive abrupt shift in baseline was found (${\hat{\Delta }}_{\mu ,2}=6.087$), with credible interindividual difference in the amount of sudden shift (i.e. $\sigma_{\Delta_{\mu ,2}}$ had an HDI outside of ROPE). Third, the group average AR parameter, $\phi_{0}$, was estimated to be slightly higher in the GCM‐RS2 than in the GCM + RS2‐IIV. Fourth, the estimated log‐odds of transitioning between regimes were notably closer to 0 in the GCM‐RS2 compared to those from the GCM + RS2‐IIV, indicating that the probabilities of staying the regimes in this model were lower compared to those from the GCM + RS2‐IIV. This characteristic of the model was a direct contributor to the lower entropy (and thus, regime clarity) of the GCM‐RS2 (.11 compared to .76 in the GCM + RS2‐IIV). Some of these discrepancies between models might be related to the omission of over‐time changes in the IIV as well as interindividual differences in over‐period slope in $\mu_{i,t}$ in the GCM + RS2.

**FIGURE 3:**
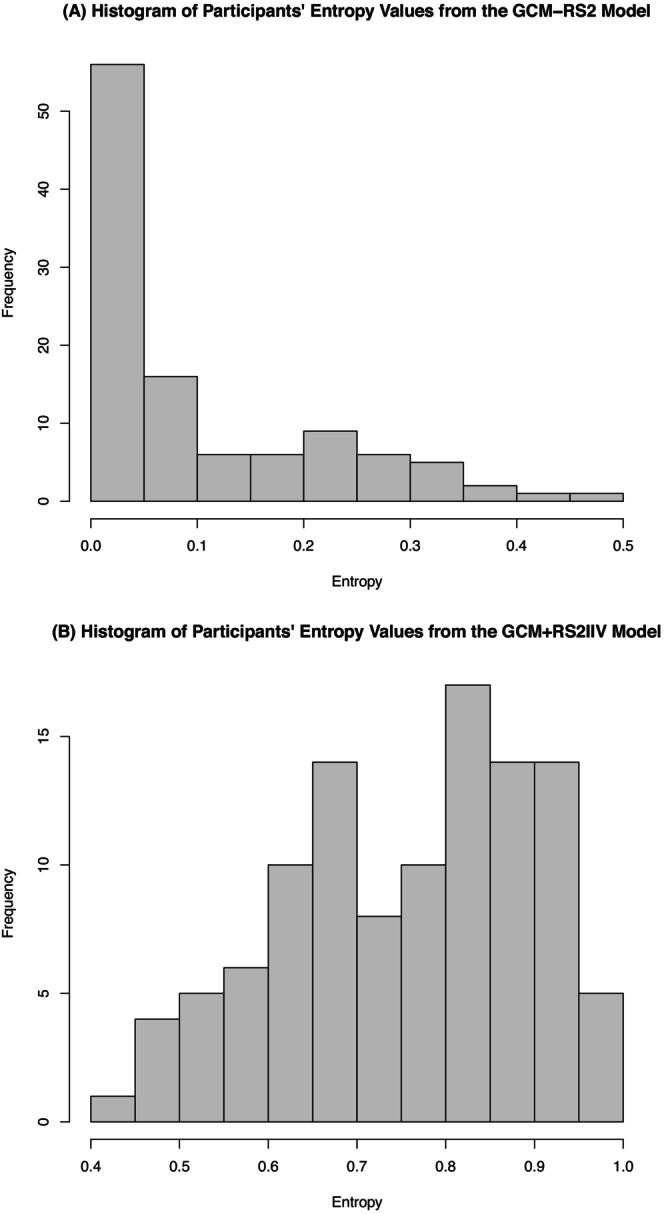
Distributions of entropy values across all participants in the empirical study (*N* = 108) from (top) the GCM + RS2 model and (bottom) the GCM + RS2‐IIV model.

To further investigate model fitting results at the individual level, we obtained the entropy value for each participant and plotted the distribution in Figure 3. We observed substantial individual differences in the entropy values and notably higher individual entropy values from the GCM + RS2‐IIV model (see plot B) than the GCM‐RS2 model (see plot A). In the former, a relatively substantial proportion (85.19%) of the participants had entropy values higher than .6 and close to half of the participants (46.30%) of the participants had entropy values that were at least .8. Thus, the GCM + RS2‐IIV considered in this empirical study produced reasonably clear regime classification for most of the participants in the study.

**FIGURE 4:**
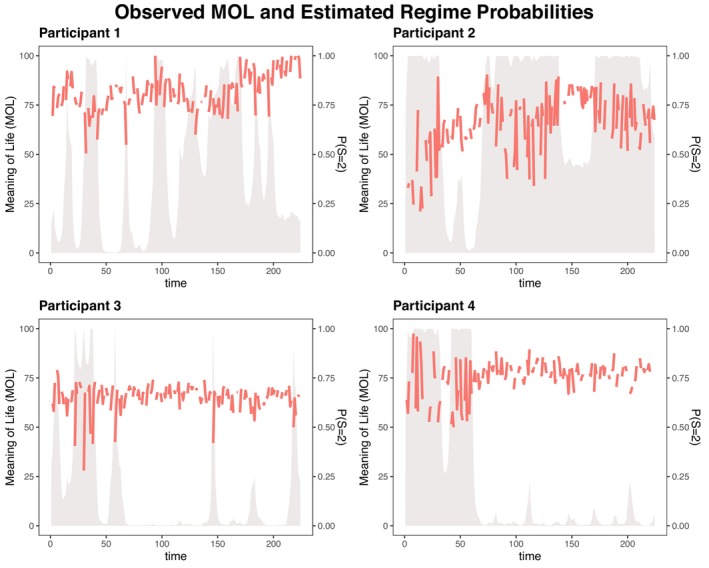
Meaning of life (MOL) dynamics (red lines) of the four participants shown in Figure 1, with model‐implied regime probabilities (shaded grey area) added based on estimation results from the GCM + RS2‐IIV. The left *y*‐axis represented the score on MOL, whereas the right *y*‐axis represented the probability of transitioning to the high‐volatility regime (i.e. the height of the shaded area), whose scale was different from the left *y*‐axis.

Results from model comparisons suggested that some but not all individuals showed abrupt changes that necessitated inclusion of the RS components. We further explored results from participants whose data motivated the development of the proposed models with gradual and abrupt changes. In Figure 4, we added the model‐implied regime probabilities, as presented in Figure 1 as the heights of the shaded regions. We observed that segments of data with high (e.g. Pr(St = 2|data)) ≥.75; see the right *y*‐axis and sustained (e.g. with more than 28 time points or 7 consecutive days) estimated probabilities of staying within the high‐volatility regime corresponded largely to data segments with surges in MOL that were not well accounted for by a linear GCM for $\mu_{i,t}$. Some examples included *t* between 120 and 170 for P1; between *t* = 60 and 140 and after t=170 for P2; and before t=56—namely, during the pre‐intervention phase—for P3 and P4. Because the model specification dictated that participants started with high probability (.9) in regime 1, subsequent transitions to a “high‐volatility” regime allowed the GCM + RS2‐IIV model to capture some participants' non‐monotonic changes (i.e. changes that reversed in direction) in MOL levels compared to their projected levels in regime 1. The extent to which the model‐implied regime probabilities mirrored fluctuations in these participants' observed MOL levels was reflected, in part, in their entropy values (P1: .43; P2: .67; P3: .70; P4: .83). Individuals with distinctly high entropy, such as P3 and P4, were indeed those who showed less frequent ongoing transitions between regimes.

In sum, the answers to the RQs can be summarized below based on the preferred GCM + RS2‐IIV. In terms of RQ1, similar to previous work utilizing non‐RS models (Li et al., 2024), we found no credible average gradual change from period to period in the baseline MOL of participants, but credible interindividual differences in the over‐period slope. For RQ2, evidence for abrupt shifts from a low‐volatility to a high‐volatility regime was found. The overall IIV level in the high‐volatility regime was 2.58 times higher than that in the low‐volatility regime. As for RQ3, no group by intervention period interaction effect was found in terms of the log‐odds of transitioning between regimes. In terms of RQ4, based on joint consideration of entropy, IC measures and computational costs, we chose the GCM + RS2‐IIV as the best‐fitting model among all candidate models. This model added a unique component to the empirical findings: the potential regime transitions in IIV for certain participants, which would have been bypassed in models without RS structures.

## 7 DISCUSSION

The present study provided an integrated framework for simultaneous representation and detection of gradual changes and regime switches in individuals' dynamics; and group‐ and individual‐level differences in these changes. Utilizing this framework, we discussed several diagnostic measures for detecting gradual and abrupt changes in dynamic features. The performance of a set of model selection metrics (e.g. IC measures, entropy, AUC and sensitivity) was evaluated in a simulation study where candidate models were fitted to simulated data with various levels of sudden shifts. Our simulation results showed that, based on the decision rule proposed in the current study, using a combination of entropy and IC measures was most effective for helping to select the best fitting model in practice. Extending results from previous studies, our empirical results suggested that there might be regime transitions in the IIV levels of MOL dynamics, and participants were more likely to transition to a high‐IIV regime later in the study that captured in part some sustained shifts in MOL levels.

Our simulation and empirical results suggested that conventional thresholds for satisfactory entropy levels such as .9 might be overly stringent for RS models with recurring transitions across regimes. We found that parameter estimation, model selection results and classification‐based performance measures such as AUC, precision and recall remained satisfactory even with entropy values that were as low as .6. Critically, entropy helped strengthen model selection results compared to only using IC‐based measures.

There are several limitations to the current work. First, our proposed models were motivated strongly by known information about our empirical study design. Hence, we built highly theory‐driven parametric models to capture the period‐to‐period changes. In more general cases where no contextual information can be used in model specifications, it is highly recommended that researchers fit a set of candidate models for TVPs (e.g. parametric, semi‐ and non‐parametric models, etc.). In addition, in the present study, we only considered linear models for gradual changes. One possible extension would be extending the linear GCM to nonlinear GCM to capture potential nonlinear changes. For example, the gradual change may be approximated by the Gompertz growth curve, where the growth rate decays exponentially over time, since participants may experience a higher rate of change during the intervention period, followed by a slower rate of change after the intervention. In a similar vein, the covariate for RS used in the empirical illustration, Period2, was coded based on the expectation that some of the most salient changes in dynamics would be manifested during the intervention period. Instead, we observed considerable heterogeneity in the timing of the sudden shifts, with some participants, such as P3 and P4, displaying sustained changes during both the intervention and post‐intervention periods. Alternative coding for Period2 may better capture such patterns of distinct dynamics.

Second, the present study assumed that the process noise terms followed normal distributions, a common assumption when applying AR‐type models. However, non‐normal process noises have been observed and well studied in economic and financial research, such as stock returns and foreign exchange rates (Bai et al., 2003). The test of the normality assumption is worthwhile in addressing concerns regarding distribution misspecifications. An alternative approach involves the semiparametric method, which uses parametric models with unspecified distributions for process noises (Wang & Zhao, 2016).

Third, in this study, the raw data were aggregated to be equally spaced to fit a discrete‐time model. However, modelling continuous‐time processes with discrete‐time models can be problematic, and continuous‐time extensions to the current model may be needed. Other possible alternatives include modelling within‐day dynamics and interpolating data to be equally spaced using cubic spline (Fisher et al., 2017) or alternative approaches. Fourth, we only considered a limited set of predictors in our empirical analysis. Although we included some targeted predictors of between‐individual and across‐phrase differences in dynamics (e.g. Treatment and Period2), other critical variables might not be included in our model. Future research should explore the inclusion of additional predictors to provide a more comprehensive understanding of the transitions between regimes. Fifth, we only included 100 MC replications in each of our simulation studies, which were limited. Future studies should consider more replications, particularly in deducing properties of the proposed modelling approach in uncertainty quantification.

It should be noted that by applying the proposed model to the empirical data, our goal was to demonstrate the ability of the proposed model and model selection metrics in capturing the change processes and selecting the most appropriate model. The modelling results did not necessarily lead to actionable intervention decisions. Translating findings on human behavioural dynamics and psychological processes into effective intervention strategies requires more rigorous work, such as experimental designs and evaluation models that take into account intervention effects that may be heterogeneous between individuals, subgroups (latent classes) and time points (see, e.g., Collins et al., 2007; Nahum‐Shani et al., 2018).

Several future directions may be pursued. First, the proposed model can potentially be extended to a multivariate model (e.g. a VAR model) to capture the relationships between variables (e.g. meaning of life and other PWB dimensions) and structural changes in these relationships over time. One challenge related to fitting such models would be the rapid increase in computational complexity caused by the increased number of model parameters, especially for models with the RS component, where the addition of a single regime would result in a substantial increase in unknown model parameters. Second, our study primarily focused on immediate effects of interventions and predictors on the outcomes. However, it is well‐known that some interventions may have delayed effects that become evident after a certain period. Future research may consider models that allow for the detection and analysis of delayed effects, which could provide a more comprehensive understanding of the underlying mechanisms of the intervention. Finally, the present study assumed data were missing at random when handling missing data in the analysis. Future work may leverage the flexibility of Bayesian missing data handling approaches by incorporating missing data models into the current modelling framework to accommodate other missing mechanisms.

It is well known that modelling the mean and variability can be confounded, as IIV may simply serve as a mechanism for absorbing “misspecification” in the mean structure (Davidian & Giltinan, 2003; Nesselroade & Salthouse, 2004). In this way, incorporating components of gradual and/or abrupt changes into IIV is not only intuitive but also offers valuable insights into potential sources of model misspecification, thereby informing future model development and refinement.

## AUTHOR CONTRIBUTIONS

**Yanling Li:** methodology; writing – original draft; formal analysis; validation. **Xiaoyue Xiong:** formal analysis; writing – review and editing; validation. **Zita Oravecz:** writing – review and editing. **Sy‐Miin Chow:** writing – review and editing; methodology; conceptualization; supervision; writing – original draft.

## CONFLICT OF INTEREST STATEMENT

The authors declare that they have no conflicts of interest.

## DISCLOSURE OF ARTIFICIAL INTELLIGENCE‐GENERATED CONTENT (AIGC) TOOLS

The authors declare that no artificial intelligence‐generated content tools were used in the preparation of this manuscript.

## Data Availability

The data that support the findings of this study are openly available at https://osf.io/vfps8/. The code for both simulation and empirical studies can be found at https://github.com/yanlingli1/gradual‐abrupt‐changes‐bayesian.

## References

[bmsp70029-bib-0001] Akaike, H. (1998). Information theory and an extension of the maximum likelihood principle. In E. Parzen , K. Tanabe , & G. Kitagawa (Eds.), Selected papers of Hirotugu Akaike (pp. 199–213). Springer.

[bmsp70029-bib-0002] Albers, C. J. , & Bringmann, L. F. (2020). Inspecting gradual and abrupt changes in emotion dynamics with the time‐varying change point autoregressive model. European Journal of Psychological Assessment, 36(3), 492–499.

[bmsp70029-bib-0003] Asparouhov, T. , & Muthén, B. (2014). Variable‐specific entropy contribution . http://www.statmodel.com/download/UnivariateEntropy.pdf

[bmsp70029-bib-0004] Bai, X. , Russell, J. R. , & Tiao, G. C. (2003). Kurtosis of GARCH and stochastic volatility models with non‐normal innovations. Journal of Econometrics, 114(2), 349–360.

[bmsp70029-bib-0005] Bradley, A. P. (1997). The use of the area under the ROC curve in the evaluation of machine learning algorithms. Pattern Recognition, 30(7), 1145–1159.

[bmsp70029-bib-0006] Bringmann, L. F. , Hamaker, E. L. , Vigo, D. E. , Aubert, A. , Borsboom, D. , & Tuerlinckx, F. (2017). Changing dynamics: Time‐varying autoregressive models using generalized additive modeling. Psychological Methods, 22(3), 409–425. 27668421 10.1037/met0000085

[bmsp70029-bib-0007] Brumback, B. A. , & Rice, J. A. (1998). Smoothing spline models for the analysis of nested and crossed samples of curves. Journal of the American Statistical Association, 93(443), 961–976.

[bmsp70029-bib-0008] Burnham, K. P. , & Anderson, D. R. (2004). Multimodel inference: Understanding AIC and BIC in model selection. Sociological Methods & Research, 33(2), 261–304.

[bmsp70029-bib-0009] Butler, J. , & Kern, M. L. (2016). The PERMA‐profiler: A brief multidimensional measure of flourishing. International Journal of Wellbeing, 6(3), 1–48.

[bmsp70029-bib-0010] Celeux, G. , & Soromenho, G. (1996). An entropy criterion for assessing the number of clusters in a mixture model. Journal of Classification, 13, 195–212.

[bmsp70029-bib-0011] Chow, S.‐M. , Grimm, K. J. , Filteau, G. , Dolan, C. V. , & McArdle, J. J. (2013). Regime‐switching bivariate dual change score model. Multivariate Behavioral Research, 48(4), 463–502. 26742002 10.1080/00273171.2013.787870

[bmsp70029-bib-0012] Chow, S.‐M. , Ho, M.‐H. R. , Hamaker, E. J. , & Dolan, C. V. (2010). Equivalences and differences between structural equation and state‐space modeling frameworks. Structural Equation Modeling, 17, 303–332.

[bmsp70029-bib-0013] Chow, S.‐M. , Ou, L. , Ciptadi, A. , Prince, E. , Hunter, M. D. , You, D. , Rehg, J. M. , Rozga, A. , & Messinger, D. S. (2018). Representing sudden shifts in intensive dyadic interaction data using differential equation models with regime switching. Psychometrika, 83(2), 476–510. 29557080 10.1007/s11336-018-9605-1 PMC7370947

[bmsp70029-bib-0014] Chow, S.‐M. , Ou, L. , Cohn, J. F. , & Messinger, D. S. (2017). Representing self‐organization and nonstationarities in dyadic interaction processes using dynamic systems modeling techniques. In A. von Davier , M. Zhu , & P. Kyllonen (Eds.), Innovative assessment of collaboration (pp. 269–286). Springer International Publishing.

[bmsp70029-bib-0015] Chow, S.‐M. , Witkiewitz, K. , Grasman, R. , & Maisto, S. A. (2015). The cusp catastrophe model as cross‐sectional and longitudinal mixture structural equation models. Psychological Methods, 20(1), 142–164. 25822209 10.1037/a0038962 PMC4506274

[bmsp70029-bib-0016] Chow, S.‐M. , You, D. , & Clouthier, T. (2020). A regime‐switching framework for formulating multi‐phase linear and nonlinear growth curves. In H. Jiao & R. W. Lissitz (Eds.), Innovative psychometric modeling and methods (pp. 193–234). Information Age Publishing.

[bmsp70029-bib-0017] Chow, S.‐M. , & Zhang, G. (2013). Nonlinear regime‐switching state‐space (RSSS) models. Psychometrika, 78(4), 740–768. 24092487 10.1007/s11336-013-9330-8

[bmsp70029-bib-0018] Chow, S.‐M. , Zu, J. , Shifren, K. , & Zhang, G. (2011). Dynamic factor analysis models with time‐varying parameters. Multivariate Behavioral Research, 46(2), 303–339. 26741330 10.1080/00273171.2011.563697

[bmsp70029-bib-0019] Chung, T. A. , & Maisto, S. A. (2006). Relapse to alcohol and other drug use in treated adolescents: Review and reconsideration of relapse as a change point in clinical course. Clinical Psychology Review, 26, 149–161. 16364524 10.1016/j.cpr.2005.11.004

[bmsp70029-bib-0020] Collins, L. M. , Murphy, S. A. , & Strecher, V. (2007). The multiphase optimization strategy (most) and the sequential multiple assignment randomized trial (smart): New methods for more potent eHealth interventions. American Journal of Preventive Medicine, 32(5), S112–S118. 17466815 10.1016/j.amepre.2007.01.022 PMC2062525

[bmsp70029-bib-0021] Davidian, M. , & Giltinan, D. M. (2003). Nonlinear models for repeated measurement data: An overview and update. Journal of Agricultural, Biological, and Environmental Statistics, 8(4), 387–419.

[bmsp70029-bib-0022] Del Negro, M. , & Otrok, C. (2008). Dynamic factor models with time‐varying parameters: Measuring changes in international business cycles . FRB of New York Staff Report (326).

[bmsp70029-bib-0023] Fahrmeir, L. , Kneib, T. , & Lang, S. (2004). Penalized structured additive regression for space‐time data: A Bayesian perspective. Statistica Sinica, 14, 715–745.

[bmsp70029-bib-0024] Fan, J. , & Zhang, W. (2008). Statistical methods with varying coefficient models. Statistics and Its Interface, 1, 179. 18978950 10.4310/sii.2008.v1.n1.a15 PMC2575822

[bmsp70029-bib-0025] Fisher, A. J. , Reeves, J. W. , Lawyer, G. , Medaglia, J. D. , & Rubel, J. A. (2017). Exploring the idiographic dynamics of mood and anxiety via network analysis. Journal of Abnormal Psychology, 126(8), 1044–1056. 29154565 10.1037/abn0000311

[bmsp70029-bib-0026] Fox, A. J. (1972). Outliers in time series. Journal of the Royal Statistical Society. Series B, Statistical Methodology, 34(3), 350–363.

[bmsp70029-bib-0027] Gates, K. M. , Chow, S.‐M. , & Molenaar, P. C. (2023). Intensive longitudinal analysis of human processes. CRC Press.

[bmsp70029-bib-0028] Grimm, K. J. , Ram, N. , & Estabrook, R. (2010). Nonlinear structured growth mixture models in Mplus and OpenMx. Multivariate Behavioral Research, 45(6), 887–909. 25419006 10.1080/00273171.2010.531230 PMC4236851

[bmsp70029-bib-0029] Haan‐Rietdijk, D. , Gottman, J. M. , Bergeman, C. S. , & Hamaker, E. L. (2016). Get over it! a multilevel threshold autoregressive model for state‐dependent affect regulation. Psychometrika, 81(1), 217–241. 25091047 10.1007/s11336-014-9417-x PMC4764683

[bmsp70029-bib-0030] Hanley, J. A. , & McNeil, B. J. (1982). The meaning and use of the area under a receiver operating characteristic (ROC) curve. Radiology, 143(1), 29–36. 7063747 10.1148/radiology.143.1.7063747

[bmsp70029-bib-0031] Hastie, T. , & Tibshirani, R. (1993). Varying‐coefficient models. Journal of the Royal Statistical Society. Series B, Statistical Methodology, 55(4), 757–779.

[bmsp70029-bib-0032] Hayes, A. M. , Laurenceau, J.‐P. , Feldman, G. , Strauss, J. L. , & Cardaciotto, L. (2007). Change is not always linear: The study of nonlinear and discontinuous patterns of change in psychotherapy. Clinical Psychology Review, 27(6), 715–723. 17316941 10.1016/j.cpr.2007.01.008 PMC3163164

[bmsp70029-bib-0033] Heiby, E. M. (1995). Chaos theory, nonlinear dynamical models, and psychological assessment. Psychological Assessment, 7(1), 5–9.

[bmsp70029-bib-0034] Henson, J. M. , Reise, S. P. , & Kim, K. H. (2007). Detecting mixtures from structural model differences using latent variable mixture modeling: A comparison of relative model fit statistics. Structural Equation Modeling: A Multidisciplinary Journal, 14(2), 202–226.

[bmsp70029-bib-0035] Heron, K. E. , & Smyth, J. M. (2010). Ecological momentary interventions: Incorporating mobile technology into psychosocial and health behaviour treatments. British Journal of Health Psychology, 15(1), 1–39. 19646331 10.1348/135910709X466063 PMC2800172

[bmsp70029-bib-0036] Hoover, D. R. , Rice, J. A. , Wu, C. O. , & Yang, L.‐P. (1998). Nonparametric smoothing estimates of time‐varying coefficient models with longitudinal data. Biometrika, 85(4), 809–822.

[bmsp70029-bib-0037] Huang, J. Z. , Wu, C. O. , & Zhou, L. (2004). Polynomial spline estimation and inference for varying coefficient models with longitudinal data. Statistica Sinica, 14, 747–772.

[bmsp70029-bib-0038] Kruschke, J. (2014). Doing Bayesian data analysis: A tutorial with R, JAGS, and Stan. Academic Press.

[bmsp70029-bib-0039] Kuppens, P. , Allen, N. B. , & Sheeber, L. B. (2010). Emotional inertia and psychological maladjustment. Psychological Science, 21(7), 984–991. 20501521 10.1177/0956797610372634 PMC2901421

[bmsp70029-bib-0040] Li, Y. , Williams, L. , Muth, C. , Heshmati, S. , Chow, S.‐M. , & Oravecz, Z. (2024). A growth of hierarchical autoregression model for capturing individual differences in changes of dynamic characteristics of psychological processes. Structural Equation Modeling: A Multidisciplinary Journal, 32, 1–14. 10.1080/10705511.2024.2402328 PMC12052329 40330774

[bmsp70029-bib-0041] Li, Y. , Wood, J. , Ji, L. , Chow, S.‐M. , & Oravecz, Z. (2022). Fitting multilevel vector autoregressive models in Stan, JAGS, and Mplus. Structural Equation Modeling: A Multidisciplinary Journal, 29(3), 452–475. 35601030 10.1080/10705511.2021.1911657 PMC9122119

[bmsp70029-bib-0042] Lu, Z.‐H. , Chow, S.‐M. , Ram, N. , & Cole, P. M. (2019). Zero‐inflated regime‐switching stochastic differential equation models for highly unbalanced multivariate, multi‐subject time‐series data. Psychometrika, 84(2), 611–645. 30859367 10.1007/s11336-019-09664-7 PMC6844193

[bmsp70029-bib-0043] Ma, J. , & Wang, T. (2004). Entropy penalized automated model selection on Gaussian mixture. International Journal of Pattern Recognition and Artificial Intelligence, 18(8), 1501–1512.

[bmsp70029-bib-0044] McKeown, G. J. , & Sneddon, I. (2014). Modeling continuous self‐report measures of perceived emotion using generalized additive mixed models. Psychological Methods, 19, 155–174. 24219542 10.1037/a0034282

[bmsp70029-bib-0045] Molenaar, P. , De Gooijer, J. G. , & Schmitz, B. (1992). Dynamic factor analysis of nonstationary multivariate time series. Psychometrika, 57(3), 333–349.

[bmsp70029-bib-0046] Molenaar, P. , Sinclair, K. O. , Rovine, M. J. , Ram, N. , & Corneal, S. E. (2009). Analyzing developmental processes on an individual level using nonstationary time series modeling. Developmental Psychology, 45(1), 260. 19210007 10.1037/a0014170

[bmsp70029-bib-0047] Nahum‐Shani, I. , Smith, S. N. , Spring, B. J. , Collins, L. M. , Witkiewitz, K. , Tewari, A. , & Murphy, S. A. (2018). Just‐in‐time adaptive interventions (JITAIs) in mobile health: Key components and design principles for ongoing health behavior support. Annals of Behavioral Medicine, 52(6), 446–462. 27663578 10.1007/s12160-016-9830-8 PMC5364076

[bmsp70029-bib-0048] Neale, M. C. , Clark, S. L. , Dolan, C. V. , & Hunter, M. D. (2016). Regime switching modeling of substance use: Time‐varying and second‐order markov models and individual probability plots. Structural Equation Modeling: A Multidisciplinary Journal, 23(2), 221–233. 26924921 10.1080/10705511.2014.979932 PMC4767507

[bmsp70029-bib-0049] Nelson, C. R. , & Plosser, C. R. (1982). Trends and random walks in macroeconmic time series: Some evidence and implications. Journal of Monetary Economics, 10(2), 139–162.

[bmsp70029-bib-0050] Nesselroade, J. R. , & Salthouse, T. A. (2004). Methodological and theoretical implications of intraindividual variability in perceptual‐motor performance. Journal of Gerontology: Psychological Sciences, 59B(2), P49–P55. 10.1093/geronb/59.2.p49 15014087

[bmsp70029-bib-0051] Ning, L. , & Luo, W. (2017). Specifying turning point in piecewise growth curve models: Challenges and solutions. Frontiers in Applied Mathematics and Statistics, 3, 19.

[bmsp70029-bib-0052] Oh, H. , Hunter, M. D. , & Chow, S.‐M. (2025). Measurement model misspecification in dynamic structural equation models: Power, reliability, and other considerations. Structural Equation Modeling: A Multidisciplinary Journal, 32(3), 511–528. 40556684 10.1080/10705511.2025.2452884 PMC12183645

[bmsp70029-bib-0053] Perron, P. (1989). The great crash, the oil price shock, and the unit root hypothesis. Econometrica: Journal of the Econometric Society, 57, 1361–1401.

[bmsp70029-bib-0054] Peter, Y. , & Jakeman, A. (1980). Refined instrumental variable methods of recursive time‐series analysis Part III. Extensions. International Journal of Control, 31(4), 741–764.

[bmsp70029-bib-0055] Röcke, C. , & Brose, A. (2013). Intraindividual variability and stability of affect and well‐being: Short‐term and long‐term change and stabilization processes. GeroPsych, 26(3), 185.

[bmsp70029-bib-0056] Schwarz, G. (1978). Estimating the dimension of a model. The Annals of Statistics, 6, 461–464.

[bmsp70029-bib-0057] Sclove, S. L. (1987). Application of model‐selection criteria to some problems in multivariate analysis. Psychometrika, 52, 333–343.

[bmsp70029-bib-0058] Segrin, C. , & Taylor, M. (2007). Positive interpersonal relationships mediate the association between social skills and psychological well‐being. Personality and Individual Differences, 43(4), 637–646.

[bmsp70029-bib-0059] Seligman, M. E. (2012). Flourish: A visionary new understanding of happiness and well‐being. Simon and Schuster.

[bmsp70029-bib-0060] Shaban, S. A. (1980). Change point problem and two‐phase regression: An annotated bibliography. International Statistical Review, 48, 83–93.

[bmsp70029-bib-0061] Shao, C. , Li, J. , & Cheng, Y. (2016). Detection of test speededness using change‐point analysis. Psychometrika, 81(4), 1118–1141. 26305400 10.1007/s11336-015-9476-7

[bmsp70029-bib-0062] Tan, X. , Shiyko, M. P. , Li, R. , Li, Y. , & Dierker, L. (2012). A time‐varying effect model for intensive longitudinal data. Psychological Methods, 17(1), 61–77. 22103434 10.1037/a0025814 PMC3288551

[bmsp70029-bib-0063] Thomas, C. , & Persons, J. B. (2013). Sudden gains can occur in psychotherapy even when the pattern of change is gradual. Clinical Psychology: Science and Practice, 20(2), 127–142.

[bmsp70029-bib-0064] Vittengl, J. R. , Anna Clark, L. , E Thase, M. , & B Jarrett, R. (2015). Detecting sudden gains during treatment of major depressive disorder: Cautions from a Monte Carlo analysis. Current Psychiatry Reviews, 11(1), 19–31. 26478724 10.2174/1573400510666140929195441 PMC4606893

[bmsp70029-bib-0065] Wang, C.‐S. , & Zhao, Z. (2016). Conditional value‐at‐risk: Semiparametric estimation and inference. Journal of Econometrics, 195(1), 86–103.

[bmsp70029-bib-0066] Wood, S. (2017). Generalized additive models: An introduction with R (2nd ed.). Chapman and Hall/CRC.

[bmsp70029-bib-0067] You, D. , Hunter, M. , Chen, M. , & Chow, S.‐M. (2019). A diagnostic procedure for detecting outliers in linear state‐space models. Multivariate Behavioral Research, 55(2), 1. 31264463 10.1080/00273171.2019.1627659 PMC6939157

